# Nox2 Modification of LDL Is Essential for Optimal Apolipoprotein B-mediated Control of *agr* Type III *Staphylococcus aureus* Quorum-sensing

**DOI:** 10.1371/journal.ppat.1003166

**Published:** 2013-02-14

**Authors:** Pamela R. Hall, Bradley O. Elmore, Cynthia H. Spang, Susan M. Alexander, Brett C. Manifold-Wheeler, Moriah J. Castleman, Seth M. Daly, M. Michal Peterson, Erin K. Sully, Jon K. Femling, Michael Otto, Alexander R. Horswill, Graham S. Timmins, Hattie D. Gresham

**Affiliations:** 1 Department of Pharmaceutical Sciences, University of New Mexico College of Pharmacy, Albuquerque, New Mexico, United States of America; 2 Research Service, New Mexico Veterans Affairs Health Care Service, Albuquerque, New Mexico, United States of America; 3 Department of Emergency Medicine, University of New Mexico, Albuquerque, New Mexico, United States of America; 4 Laboratory of Human Bacterial Pathogenesis, National Institute of Allergy and Infectious Diseases, The National Institutes of Health, Bethesda, Maryland, United States of America; 5 Department of Microbiology, Roy J. and Lucille A. Carver College of Medicine, University of Iowa, Iowa City, Iowa, United States of America; 6 Department of Internal Medicine, University of New Mexico School of Medicine, Albuquerque, New Mexico, United States of America; Columbia University, United States of America

## Abstract

*Staphylococcus aureus* contains an autoinducing quorum-sensing system encoded within the *agr* operon that coordinates expression of virulence genes required for invasive infection. Allelic variation within *agr* has generated four *agr* specific groups, *agr* I–IV, each of which secretes a distinct autoinducing peptide pheromone (AIP1-4) that drives *agr* signaling. Because *agr* signaling mediates a phenotypic change in this pathogen from an adherent colonizing phenotype to one associated with considerable tissue injury and invasiveness, we postulated that a significant contribution to host defense against tissue damaging and invasive infections could be provided by innate immune mechanisms that antagonize *agr* signaling. We determined whether two host defense factors that inhibit AIP1-induced *agr*I signaling, Nox2 and apolipoprotein B (apoB), also contribute to innate control of AIP3-induced *agr*III signaling. We hypothesized that apoB and Nox2 would function differently against AIP3, which differs from AIP1 in amino acid sequence and length. Here we show that unlike AIP1, AIP3 is resistant to direct oxidant inactivation by Nox2 characteristic ROS. Rather, the contribution of Nox2 to defense against *agr*III signaling is through oxidation of LDL. ApoB in the context of oxLDL, and not LDL, provides optimal host defense against *S. aureus agr*III infection by binding the secreted signaling peptide, AIP3, and preventing expression of the *agr*-driven virulence factors which mediate invasive infection. ApoB within the context of oxLDL also binds AIP 1-4 and oxLDL antagonizes *agr* signaling by all four *agr* alleles. Our results suggest that Nox2-mediated oxidation of LDL facilitates a conformational change in apoB to one sufficient for binding and sequestration of all four AIPs, demonstrating the interdependence of apoB and Nox2 in host defense against *agr* signaling. These data reveal a novel role for oxLDL in host defense against *S. aureus* quorum-sensing signaling.

## Author Summary

*Staphylococcus aureus* is a common colonizer of humans but can also cause severe, invasive infection. *S. aureus* uses a secreted peptide-based communication system, *agr*, to induce production of virulence factors needed for invasive infection. Allelic variation has generated four *agr* types, *agr* I–IV, and each secretes a distinct autoinducing peptide (AIP1-4) that differs in amino acid sequence and length. Understanding host factors that prevent signaling by each of the four *agr* specific groups (*agr*I–IV) could provide opportunities for prevention of infection or therapeutic intervention. We previously demonstrated that apolipoprotein B (apoB), the major structural protein of very low and low density lipoproteins (VLDL, LDL), binds to the secreted *agr*I peptide, AIP1, and prevents *agr* signaling. In addition, the NADPH oxidase Nox2 produces reactive oxygen species which directly modify and inactive AIP1. Here we examined the role of apoB and Nox2 in defense against *agr*III-signaling. We found that apoB in oxidized LDL, but not in native LDL, mediated optimal binding of AIP3. Also, unlike AIP1, Nox2 did not directly inactivate AIP3. Rather Nox2 contributed to defense against *agr*III-signaling by oxidizing LDL. Furthermore, we found that oxLDL bound all four AIPs and antagonized *agr* signaling by each *agr* allele *in vitro*. These results expand our understanding of host defense against *S. aureus agr* signaling.

## Introduction

*Staphylococcus aureus* uses global gene regulation to coordinate gene transcription required for survival within distinct host niches [1]–[10]. One of these global regulators is a four gene operon, *agr*, that encodes a quorum sensing system that combines secretion of an autoinducing peptide pheromone (AIP) with a sensor regulator. Activation of this system upregulates genes for toxins, hemolysins, lytic enzymes, and metabolic pathways that are required for a phenotypic change in this pathogen from an adherent colonizing phenotype to one associated with significant tissue injury and invasiveness [11]–[13]. *agr* upregulated virulence factors are associated with acute infections particularly at epithelial barriers like the skin and lung [13]–[18] and induce death and dysfunction of phagocytic and epithelial cells responsible for bactericidal clearance [10], [19]–[23]. Therefore, a significant contribution to host defense against tissue damaging and invasive infections at these sites could be provided by innate immune mechanisms that antagonize *agr* signaling thus permitting optimal phagocyte and epithelial cell function. While the molecular mechanisms involved in *agr* sensing and signaling have been extensively studied [11], the mechanisms by which host innate barriers antagonize *agr* sensing to control tissue damage and cell injury have not been fully elucidated [24]–[29]. In this regard, we previously reported that both the Nox2 NADPH oxidase and the major structural protein of very low and low density serum lipoproteins (VLDL, LDL), apolipoprotein B (apoB), antagonize AIP dependent activation of its cognate receptor within the *agr*I allele [24], [27]. Because three additional *agr* alleles are represented within the species *S. aureus* and because all four alleles are associated with significant disease in humans [30], [31], we postulated that either or both of these innate immune barriers could be important for antagonism of signaling by other *agr* types.

Each *agr* allele encodes a unique secreted AIP that differs in amino acid content and length but contains a common thiolactone bond that creates a 5-membered ring essential for biologic function [11]. Importantly, secretion of AIP represents an opportunity for host or environmental control of *agr* signaling by either direct modification of key amino acids, cleavage of the thiolactone bond, proteolytic degradation, or sequestration to prevent AIP binding to its receptor, AgrC. For AIP1, reactive oxygen species (ROS) generated by the Nox2 NADPH oxidase expressed in phagocytes and other cells directly modify a key C-terminal member of the thiolactone ring, a methionine, to form methionine sulfoxide [27]. While retaining its cyclic structure, this modification is sufficient to render AIP1 biologically inactive. In addition, the large structural protein of serum LDL, apoB, binds directly to cyclic AIP1, but not its inactive linear form, preventing its activation of its cognate receptor AgrC [24]. Importantly, loss of either Nox2 or apoB in the form of LDL is sufficient to promote *agr*I-mediated invasive infection beyond epithelial and mucosal barriers. To extend these studies to the other *agr* alleles, we first focused on the contribution of these two barriers to host antagonism of *agr*III signaling. AIP3 is shorter and composed of amino acids that are more resistant to oxidant modification as compared to AIP1 and therefore might be less amenable to control by either apoB or ROS. We hypothesized that Nox2 and apoB within the context of its serum lipoproteins would differ in the molecular mechanism by which they antagonize *agr*III signaling and that this difference would impact the susceptibility to invasive infection in mouse models that lack these innate barriers.

Here, we show that optimal host innate defense against *agr*III-mediated signaling requires binding and sequestration of AIP3 by apoB, not in the form of LDL but in LDL oxidized by Nox2. Importantly, these studies revealed an important role for oxLDL in binding to and antagonizing signaling by all four *agr* alleles. In addition, while ROS directly inactivate AIP1 and 4, they do not affect the biologic function of either AIP 2 or 3. Thus, the contribution of Nox2 in antagonizing *agr* signaling for these alleles is primarily through the production of oxLDL. OxLDL-mediated antagonism of *agr* signaling inhibits *agr* driven virulence factor expression by all four alleles, providing a mechanistic basis for its importance in preventing invasive *S. aureus* infection which we demonstrate in a murine *agr*III-mediated skin infection model. Therefore, while best studied for its contribution to atherosclerosis, our data reveal a novel role for oxLDL as a host defense effector that controls *S. aureus agr*-mediated signaling.

## Results

### Nox2 oxidation of LDL is required for optimal apolipoprotein B-mediated antagonism of *agr*III-signaling

**Figure 1:**
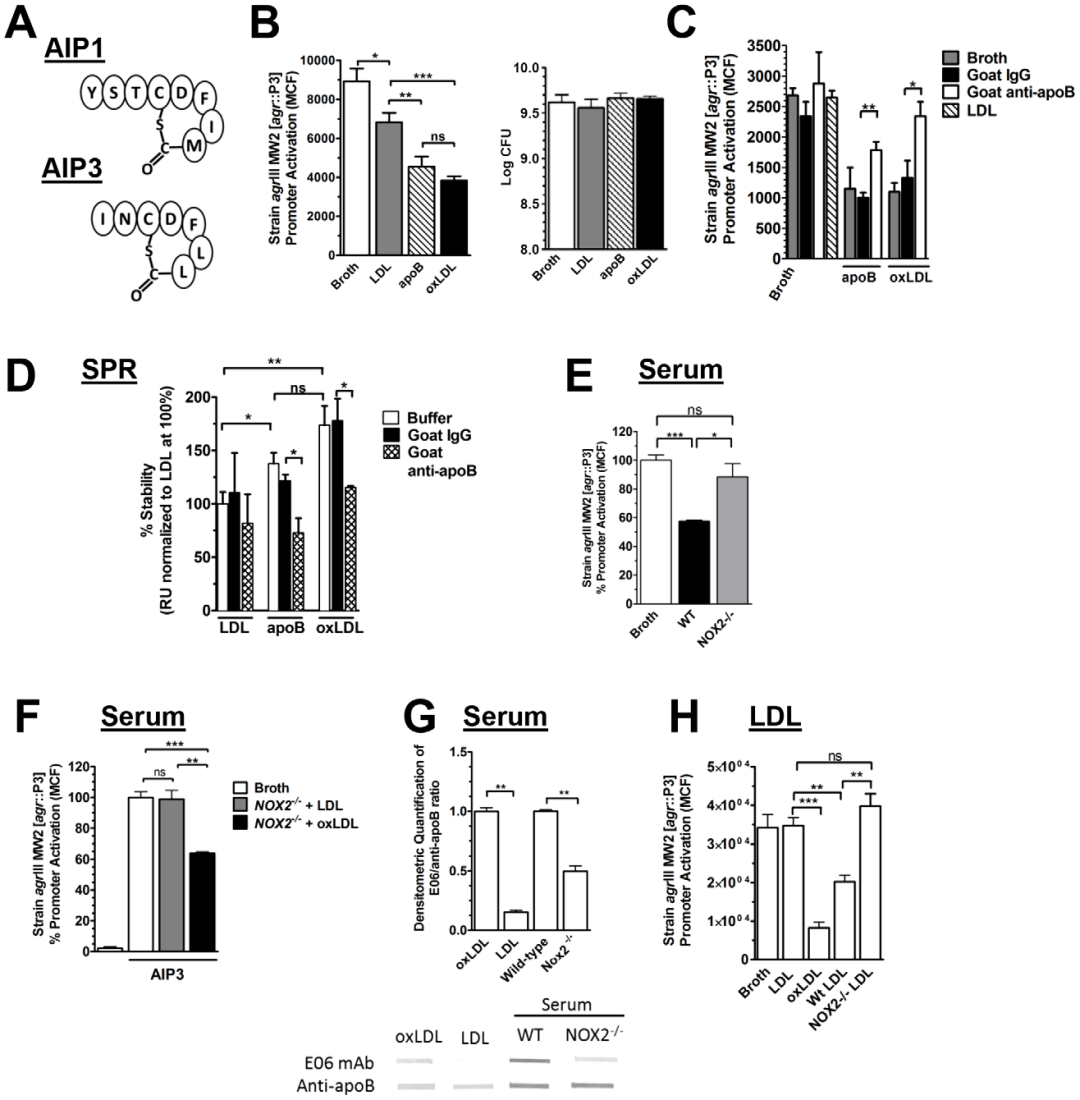
Optimal apolipoprotein B-mediated antagonism of *agr*III-signaling requires oxidation of LDL via Nox2. (A) Schematic representation of AIP1 and AIP3. (B) Strain *agr*III MW2 [*agr*::P3-yfp] was cultured overnight with broth control or 10 nM LDL, apoB or oxLDL, and *agr*::P3 activation was measured by flow cytometry. Colony forming units (cfus) were determined by plating on sheep blood agar. (C) Anti-apoB antibody reverses antagonism of *agr*::P3 promoter activation in MW2. Strain *agr*III MW2 [*agr*::P3-yfp] was cultured overnight with broth control, 10 nM apoB or oxLDL, along with 30 nM control IgG or 30 nM apoB-specific IgG. (D) LDL, ApoB or oxLDL binding to immobilized AIP3 was measured by SPR following lipoprotein incubation with apoB-specific IgG or control IgG. Data were normalized to the mean ± SEM of LDL binding in the absence of antibody. (E) Blood was collected from wild-type or *Nox2^−/−^* mice. After clearing, the serum was heat inactivated and diluted to 10% in TSB for overnight culture with strain *agr*III MW2 [*agr*::P3-yfp]. *agr*::P3 promoter activation was measured by flow cytometry. Data reported are the mean ± SEM normalized to broth control. (F) Strain *agr*III MW2 [*agr*::P3-yfp] cultured 4 h with 100 nM AIP plus 10% sera from *Nox2^−/−^* mice along with 50 nM LDL or 50 nM oxLDL. Data points represent the mean ± SEM normalized to broth control. (G) Immunoblot detection of apoB and oxidized LDL. Control LDL and oxLDL plus serum from wild-type and *Nox2^−/−^* mice were vacuum transferred to nitrocellulose and stained for oxLDL using monoclonal antibody E06 or rabbit polyclonal antibody to apoB. A representative blot is shown. Band intensity was quantified using Carestream Molecular Imaging software (New Haven, Connecticut), and data normalized to oxLDL or wild-type sera with E06/apoB ratios equal to 1. (H) Strain *agr*III MW2 [*agr*::P3-yfp] was cultured overnight with exogenous AIP3 (50 nM) and 10 nM of either human LDL, human oxLDL, LDL purified from wild-type mice, LDL purified from *Nox2^−/−^* mice or broth control. ns, not significant; *, p<0.05; **, p<0.01; ***, p<0.001.

Innate immunity controls *agr*I-mediated invasive *S. aureus* infection by early extravasation of activated neutrophils and apoB-containing plasma lipoproteins that act to antagonize *agr*I signaling [24], [27]. Induction of *agr* signaling requires AIP cyclized through a 5-membered thiolactone ring. AIP binds and activates AgrC which generates phosphorylated AgrA. Phosphorylated AgrA activates the *agr* P3 promoter generating the effector molecule RNAIII. ApoB recognizes the cyclic, active form of AIP1 and prevents binding to and signaling through its cognate AgrC receptor [24]. Whereas AIP1 and AIP3 differ in amino acid sequence and in length of the amino terminal tail, they both contain the thiolactone ring (Fig. 1A). We postulated that apoB would antagonize AIP3-dependent activation of AgrC, but that conformational changes of apoB might be required for optimal antagonism of the smaller peptide pheromone of the *agr*III strains. In addition to binding the *agr* P3 promoter, phosphorylated AgrA directly enhances transcription of genes encoding phenol soluble modulin virulence factors (PSM alpha, PSM beta) [32]–[34]. ApoB alone significantly inhibited transcription of *psm*α in clinical isolates USA300 LAC (*agr*I) and USA400 MW2 (*agr*III) [32], [35]–[37], whereas LDL at equivalent apoB concentration did not inhibit *psmα* transcription in the *agr*III strain MW2 (Fig. S1A). The ability of apoB alone to inhibit *agr*III-mediated signaling independent of LDL suggests that the conformation of apoB in LDL is not optimal for binding AIP3. Oxidation of LDL is known to alter the conformation of apoB within the lipoprotein particle [38]–[40]. We predicted that apoB present in oxLDL would significantly antagonize *agr*III-mediated signaling compared to LDL. Using strain *agr*III MW2 ([*agr*::P3-yfp] in which activation of the *agr*::P3 promoter drives expression of YFP [41], both apoB alone and oxLDL at equivalent apoB concentration significantly antagonized *agr*::P3 promoter activation as compared to LDL (Fig. 1B). These results were not due to lipoprotein effects on bacterial viability as demonstrated by the number of colony forming units (cfus). Intriguingly, LDL and oxLDL were equally efficacious in antagonism of AIP1 dependent signaling (Fig. S1B), indicating that apoB within either particle is sufficient to neutralize AIP1. To confirm that apoB, and not a lipid component of oxLDL was responsible for antagonizing AIP3-mediated signaling, we determined the effects of antibodies against apoB on the ability of apoB and oxLDL to inhibit *agr*III-dependent signaling. Pre-treatment of apoB and oxLDL with an antibody specific to a linear peptide within apoB, but not with an isotype control antibody, significantly inhibited apoB-mediated antagonism of *agr*::P3 promoter activation in this *agr*III strain (Fig. 1C). Thus apolipoprotein B is sufficient to antagonize *agr*III-dependent signaling and optimal antagonism requires apoB presented in the context of oxLDL rather than LDL.

ApoB antagonism of *agr* type I-mediated virulence entails direct binding to AIP1 [24], and based on the results of our signaling assays, we predicted that oxLDL would bind AIP3 at significantly higher levels relative to LDL. We analyzed binding of LDL, apoB and oxLDL, at equivalent apoB concentrations to immobilized AIP1 and AIP3 via Surface Plasmon Resonance (SPR). As expected, oxLDL and apoB bound at significantly higher levels to cyclic AIP3 as compared to LDL (Fig. 1D). In contrast, all 3 bound equally to AIP1 (Fig. S1C). These data support our results on the requirement of oxLDL for antagonism of AIP3-dependent signaling, whereas LDL alone was sufficient to antagonize AIP1 signaling. None of the 3 lipoproteins bound significantly to linear AIP3 (L-AIP3) (data not shown), proving that apoB recognition of AIP3 requires the active, cyclic conformation of AIP3. Furthermore, AIP3 binding by apoB and oxLDL was significantly inhibited by antibody specific to a linear peptide within apoB but not control antibody (Fig. 1D), indicating that apoB is responsible for binding of oxLDL to AIP3. The ability of the anti-peptide antibody to reverse functional antagonism in the reporter assay and to block AIP3 binding by both apoB and oxLDL, but not LDL, suggests a conformation-dependent AIP3 binding site within apoB that is also present in oxLDL but absent in LDL. Therefore, the mechanism by which oxLDL antagonizes *agr*III signaling includes enhanced binding of AIP3 relative to LDL, and this binding is mediated by apoB and not the lipid components of oxLDL.

Although AIP expressed from each of the four *agr* alleles differ in amino acid sequence and length, the peptides share a common five-membered thiolactone bond. To determine whether oxLDL universally binds *S. aureus* AIPs and antagonizes signaling by each of the four *agr* alleles, we also measured oxLDL binding to AIP2 and AIP4 (Figure S1D,E) by SPR. OxLDL bound immobilized AIP2 and AIP4, but not the inactive linear peptides, in a dose-dependent manner again illustrating the necessity of the thiolactone bond for apoB-dependent binding. Furthermore, oxLDL antagonized *agr*::P3 promoter activation by both an *agr*II (AH430 – SA502A) and *agr*IV (AH1872 – MN TG) clinical isolate (Fig. S1F). These data suggest that oxLDL could act as a universal innate inhibitor of *agr*-signaling mediated by each of the four *S. aureus agr* alleles.

ROS production by the NADPH oxidase Nox2 is essential for control of invasive *S. aureus* infection [42]–[44], and facilitates oxidation of LDL [45], [46]. We postulated that Nox2 would contribute to apoB-mediated control of *agr*III *S. aureus* virulence by oxidation of LDL. If correct, LDL from the serum of *Nox2* knockout mice [42] would primarily be in the form of native LDL whereas LDL from the serum of wild-type mice would include oxLDL and would better inhibit *agr*III-signaling relative to LDL from *Nox2^−/−^* mice. We examined serum from wild-type and *Nox2^−/−^* mice for antagonism of *agr*::P3 promoter activation in the *agr*III isolate MW2 and for the presence of LDL and oxLDL. As predicted, serum from wild-type mice, but not from *Nox2^−/−^* mice, significantly antagonized *agr*::P3 promoter (Fig. 1E), and addition of oxLDL, but not LDL, to serum from *Nox2^−/−^* mice restored *agr*III-antagonism to wild-type levels (Fig. 1F). Relative serum levels of oxLDL and LDL from wild-type and *Nox2^−/−^* mice were determined by immunoblot analysis using an antibody against apoB and a monoclonal antibody (E06) which detects epitopes present in oxLDL but absent in LDL [47], [48]. Serum from wild-type mice had a significantly higher E06/apoB ratio compared to *Nox2^−/−^* mice (Fig. 1G), indicating that serum from wild-type mice contains more oxLDL than serum from *Nox2^−/−^* mice. Although significantly reduced compared to wild-type mice, serum of *Nox2^−/−^* mice had some E06 reactivity, indicating the presence of small amounts of oxLDL resulting from oxidation mechanisms distinct from Nox2. Interestingly, the amount of E06 positivity in the LDL control fraction reflects a nominal oxidation level in LDL preparations that may vary by preparation, method and storage. This variable level of nominal oxidation likely resulted in the small but significant LDL antagonism of *agr*III-signaling observed in some experiments (Fig. 1B). To prove that apoB-containing lipoprotein oxidized by Nox2 is the serum component from wild-type mice responsible for blocking *agr*III activation, we measured the ability of LDL purified from the serum of wild-type versus *Nox2^−/−^* mice to antagonize *agr*III-dependent quorum-sensing signaling. At equivalent protein concentrations, LDL purified from the serum of *Nox2^−/−^* mice did not inhibit *agr*::P3 promoter activation in the *agr*III isolate MW2 while LDL from wild-type mice significantly inhibited *agr*III signaling (Fig. 1H). There was no significant inhibition of *agr*::P3 promoter activation in the presence of either *Nox2^−/−^* LDL or our human LDL control with minimal EO6 positivity, further demonstrating the requirement for oxLDL in *agr*III-antagonism. In addition, purified LDL from wild-type mice had a 30% greater lipid oxidation level compared to LDL from *Nox2^−/−^* mice (Fig. S2A). Thus, the ability of serum to control *agr*III-signaling *in vitro* is dictated by the presence of oxLDL within that serum and Nox2 is a major contributor to oxidation of serum LDL. These data indicate that Nox2 contributes to host control of *agr*III-mediated quorum sensing in part via oxidation of LDL that induces a conformational change in apoB required for optimal AIP3 binding and sequestration.

### Reduction of serum apoB levels in *Nox2* knockout mice but not wild-type mice significantly increases susceptibility to *agr*III *S. aureus* invasive infection

**Figure 2:**
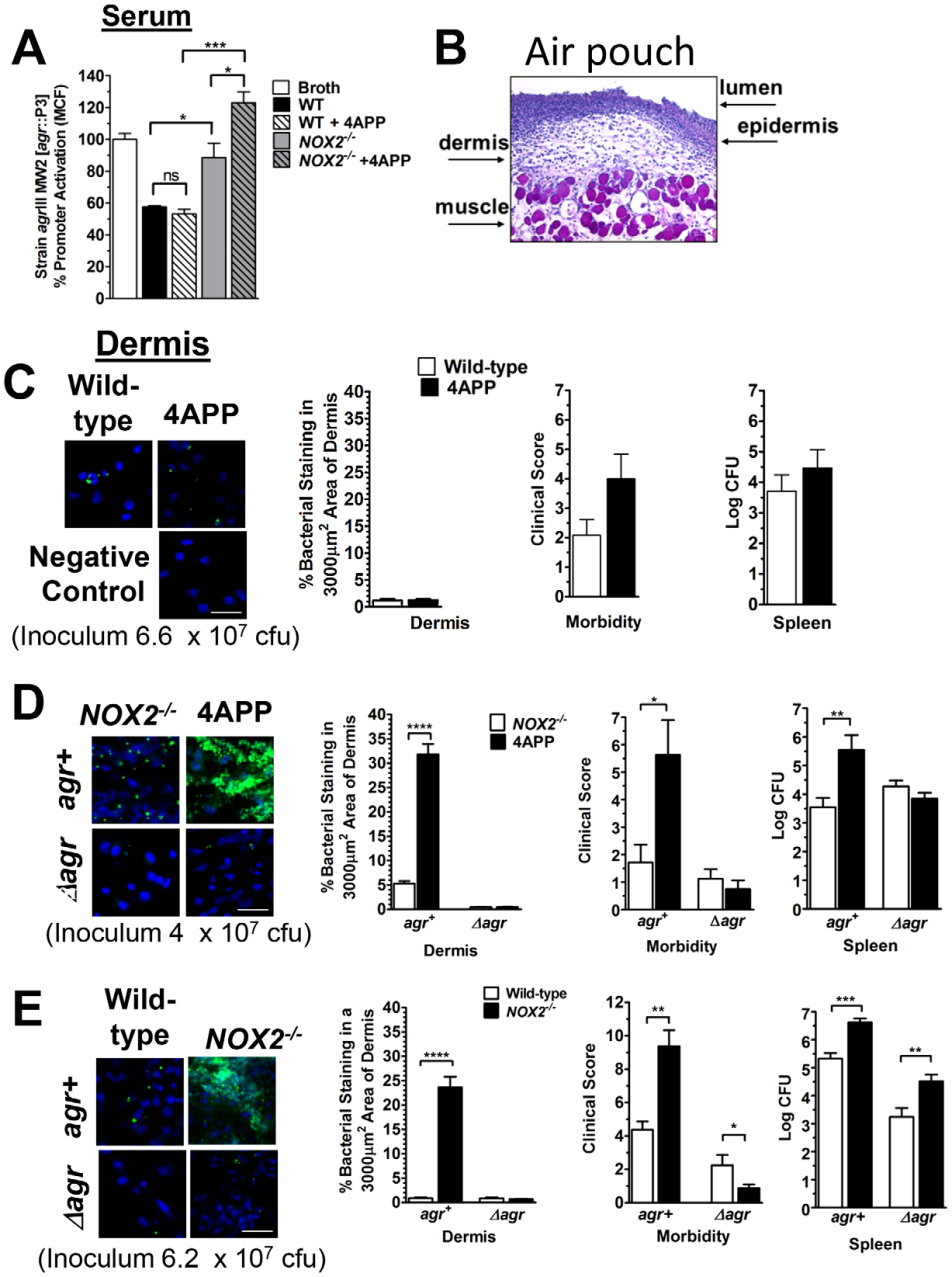
Reduction of serum apoB levels in *Nox2* knockout mice but not wild-type mice significantly increases susceptibility to *agr*III-mediated *S. aur*eus virulence. (A) Blood was collected from wild-type or *Nox2^−/−^* mice treated with 4APP or vehicle control. After clearing, the serum was heat inactivated and diluted to 10% in TSB for overnight culture with *agr*III MW2 [*agr*::P3-yfp]. *agr*::P3 promoter activation was measured by flow cytometry. Data reported are the mean ± SEM normalized to broth control at 100%. (B) Air-pouch schematic showing locations of lumen, epidermis, dermis and muscle. (C–E) Air-pouches were generated on the backs of 8 to 12 week old wild-type or *Nox2^−/−^* mice. Where indicated 4APP treated mice were injected i.p. with 100 µl of 5 mg/ml 4APP or vehicle control at 48 h and 24 h prior to infection with MW2 or its *agr* deletion mutant (Δ*agr*) at the indicated concentrations. At time zero, air-pouches were injected with early exponential phase MW2. At 28 h post-infection, the following parameters were determined and data reported as the mean ± SEM: (Left to right) Representative confocal images of dermis from indicated pouches stained with TO-PRO-3 (Invitrogen; blue) and anti-*S. aureus* antibody (green fluorophore) (Scale bar = 20 µm); Quantification of bacterial density in dermis of pouches; Morbidity was scored on a 0–14 point scale and was based on weight loss, appearance, level of dehydration, mobility and responsiveness; Bacterial burden (Log CFU) in spleen. (C) Wild-type mice treated with 4APP or vehicle control. (D) *Nox2^−/−^* mice treated with 4APP or vehicle control. (E) Wild-type and *Nox2^−/−^* mice. *, p<0.05; **, p<0.01; ***, p<0.001; ****, p<0.0001.

Virulence factors regulated by *agr* are essential for invasive skin infection and invasion of bacteria from the host epidermis into the underlying dermis [13], [16], [18], [24]. Having demonstrated that the ability of serum to control *agr*III-signaling is attributable to oxLDL, we postulated that the ability of serum to antagonize quorum sensing would predict *in vivo* susceptibility to *agr*III-invasive infection. We used the drug 4APP, which significantly reduces serum apoB levels by inhibition of lipoprotein secretion from the liver, to reduce apoB [49], [50] in wild-type and *Nox2^−/−^* mice prior to infection with the *agr*III strain MW2. Although 4APP treatment decreased serum levels of apoB-containing lipoproteins by 49% compared to buffer controls (Fig. S2B), serum from these mice was as effective as serum from vehicle treated mice in inhibiting *agr*::P3 promoter activation in MW2 (Fig. 2A) suggesting that 4APP treatment alone did not significantly affect levels of oxLDL. To confirm this, we measured lipid oxidation of purified LDL from the 4APP- or buffer control-treated wild-type mice. At equivalent protein concentrations, LDL purified from the 4APP treated wild-type mice had an almost 2-fold greater level of lipid oxidation compared to LDL from vehicle treated wild-type mice (Fig. S2C). Therefore, although the concentration of LDL was reduced by 49% in serum of 4APP treated mice, the overall concentration of oxLDL was not significantly affected, resulting in equivalent serum antagonism of *agr*III-signaling. In contrast, serum from *Nox2^−/−^* mice, that contains significantly reduced levels of oxLDL compared to wild-type mice (Fig. 1G), was not optimal for inhibition of *agr*::P3 promoter activation in the *agr*III isolate MW2 and serum from 4APP-treated *Nox2^−/−^* mice was even less effective (Fig. 2A). This suggests that loss of LDL oxidized by both Nox2 and other mechanisms results in the greatest impairment in *agr*III-antagonism.

Treatment of wild-type mice with 4APP reduced plasma levels of LDL (Fig. S2B) but did not alter the ability of the mice to oxidize LDL still being secreted from the liver (Fig. S2C). Because serum from vehicle and 4APP treated wild-type mice had similar levels of oxLDL and equally antagonized *agr*III-signaling *in vitro* (Fig. 2A), we predicted that 4APP treatment alone would not increase the susceptibility of wild-type mice to invasive MW2 infection. Using an air-pouch model of infection [24], [27], [28], [51]–[54] to determine *agr*III-dependent invasion of bacteria from the epidermis into the dermis (Fig. 2B), we infected wild-type mice treated with either 4APP or vehicle with a dose of MW2 that wild-type mice are able to maintain at the epithelial barrier. There was no increased susceptibility of the 4APP treated mice to this dose (Fig. 2C). These results confirmed our *in vitro* data that the level of oxLDL in these mice was sufficient to prevent *agr*III-quorum sensing required for invasive infection. These data demonstrate that whereas reduction of LDL in the serum of Nox2 competent mice is sufficient to increase susceptibility to *agr* type I *S. aureus* infection [24], it does not increase susceptibility to *agr*III invasive infection because oxLDL levels are sufficient for protection.

Our serum data indicate that in the absence of Nox2, the resulting reduction of oxLDL significantly impairs apoB-mediated antagonism of *agr*III-dependent quorum sensing indicating that apoB within oxLDL is primarily responsible for suppressing *agr*III signaling (Fig. 2A). Therefore, we predicted that 4APP treatment of mice lacking Nox2 would significantly increase susceptibility to *agr*III-dependent MW2 invasive infection beyond the loss of Nox2 alone. Because *Nox2^−/−^* mice are highly susceptible to *S. aureus* infection due to the critical role of reactive oxidants in host defense against this pathogen [42], and because we predicted that susceptibility would be further increased following 4APP treatment, a reduced inoculum of MW2 was used in these experiments (Fig. 2D) relative to the other infection studies (Fig. 2C,E). As predicted, Nox2 knockout mice treated with 4APP prior to infection with MW2 had significantly increased bacterial invasion into the dermis, morbidity and bacterial burden in the spleen compared to control treated *Nox2^−/−^* mice (Fig. 2D). Moreover, *Nox2^−/−^* mice treated with 4APP were not more susceptible to infection with an isogenic *agr* deletion mutant of MW2 (Δ*agr*) compared to controls, confirming that the contribution of Nox2 to apoB-mediated host defense is specific to control of *agr*-mediated pathogenesis. In contrast, infection of *Nox2^−/−^* mice with MW2 at an inoculum readily controlled by wild-type mice resulted in significant dermal invasion, morbidity and bacterial burden in the spleen as compared to wild-type mice. In addition, infection with the MW2 *agr* deletion mutant also resulted in increased dissemination to the spleen (Fig. 2E). Therefore, unlike the *agr*-specific role of apoB, the protective role of Nox2 is not limited to control of *agr-*mediated virulence but has broader implications for host antibacterial defense such as contributing to direct killing of bacteria in the spleen [46], [55]–[57].

These data confirm that apoB-mediated serum antagonism of *agr*III-signaling *in vitro* is a predictor of *in vivo* susceptibility to *S. aureus agr*III-dependent invasive infection. In addition, the increased susceptibility to *agr*III-mediated invasive infection following reduction of serum apoB is dependent upon the presence or absence of Nox2, indicating a novel role for Nox2 in host defense against *agr*III-dependent infection by promoting apoB-mediated antagonism of quorum sensing. Whereas reduction of apoB in the context of LDL is not sufficient to increase susceptibility to *agr*III-mediated invasive infection, reduction of apoB in the form of oxLDL is sufficient.

### oxLDL antagonizes *agr*III-mediated virulence factor transcription and expression in MRSA and MSSA clinical isolates

**Figure 3:**
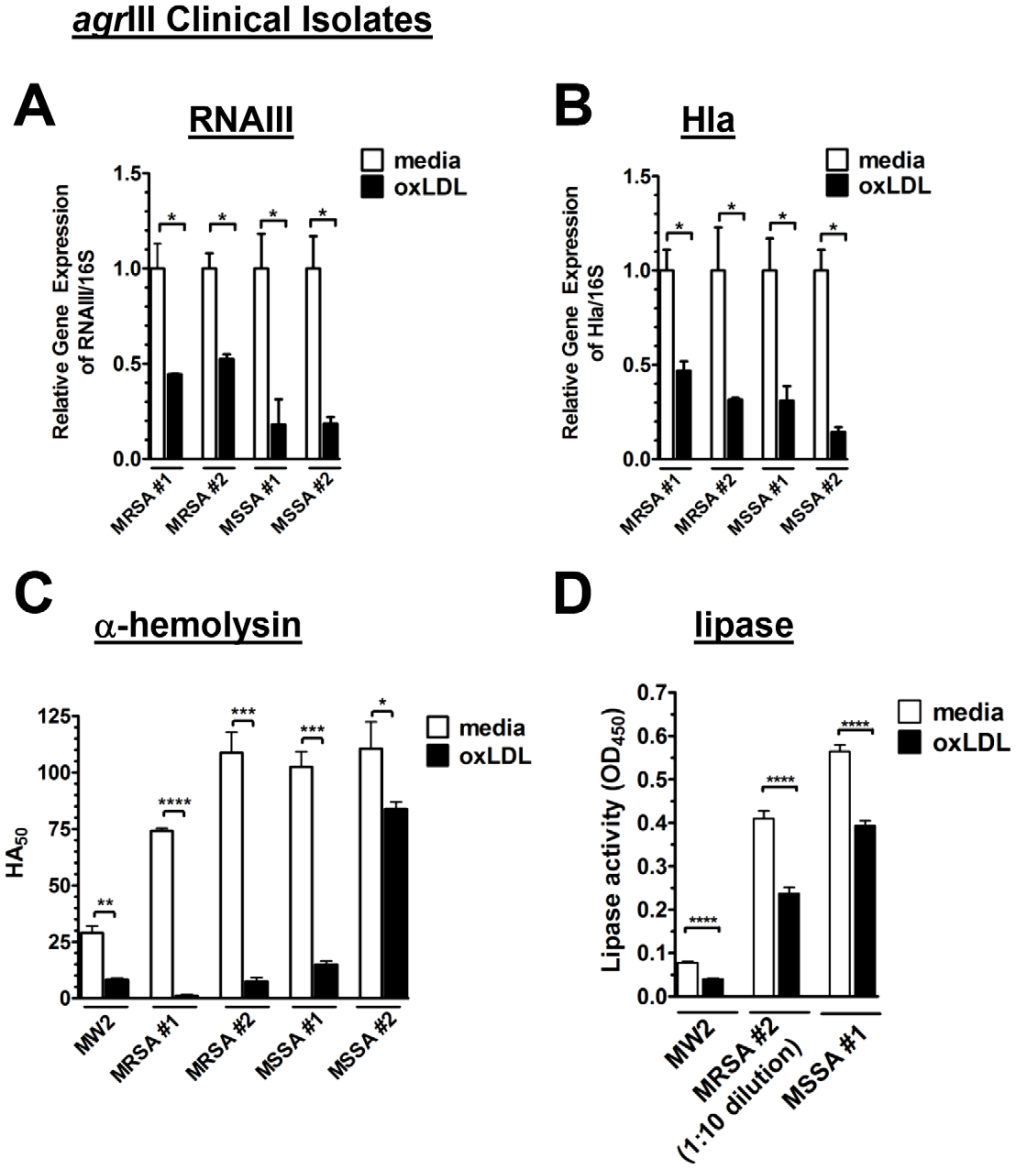
Effect of oxLDL on antagonism of *agr*III dependent virulence factor production by MRSA and MSSA clinical isolates. (A–D) Bacteria were cultured overnight in the presence of media control or oxLDL (50 nM). (A) RNAIII and (B) *hla* transcription relative to 16S rRNA was determined by qRT-PCR (C) Alpha-hemolysin content of culture supernatants was measured by the ability to lyse rRBCs. (D) Supernatants from (C) were also assessed for lipase activity determined by rate of cleavage of the triglyceride substrate tributyrin. Data reported as the mean ± SEM. *, p<0.05; **, p<0.01; ***, p<0.001; ****, p<0.0001.

Quorum-sensing through *agr* regulates expression of over 100 genes, many of which encode virulence factors necessary for invasive infection [11], [13], [58]. To confirm that the role of apoB extends to *agr*III-dependent transcription and translation of virulence factors and that this protection was not limited to isolate MW2, we first determined whether oxLDL inhibited *agr*III-dependent transcription by randomly selected MRSA and methicillin sensitive *S. aureus* (MSSA) *agr*III clinical isolates. OxLDL significantly inhibited transcription of both the *agr* effector molecule RNAIII and of a key *agr* regulated virulence factor, alpha hemolysin (Hla), by both MRSA and MSSA *agr*III clinical isolates (Fig. 3A,B). Likewise, oxLDL significantly inhibited production of Hla as determined by functional assay (Fig. 3C). This effect was not due to direct lipoprotein interaction with Hla because neither LDL nor oxLDL reduced the activity of purified Hla (Fig. S3A). Although two of the selected clinical isolates failed to produce the virulence factor staphylococcal lipase, oxLDL again significantly inhibited *agr*III-dependent lipase secretion as determined by functional assay of the remaining isolates (Fig. 3D). The decrease in lipase activity was not due to direct inhibition by the lipoproteins (Fig. S3B). These results extend our observations of oxLDL-mediated control of *agr*III-signaling and invasive infection to virulence factor expression by current clinical isolates of both MRSA and MSSA indicating that the contribution of oxLDL as a barrier to *agr*III infection is not limited to a single genetic background of the pathogen.

AIP binding and *agr*::P3 promoter activation analyses suggest that oxLDL would also inhibit *agr*-dependent transcription and virulence factor translation by *agr*I, *agr*II and *agr*IV MRSA and MSSA clinical isolates. As predicted, oxLDL antagonized *agr*-dependent transcription of RNAIII and hla along with production of functional Hla in clinical isolates representing *agr*I, *agr*II and *agr*IV alleles (Fig. S3C–E). Therefore, oxLDL can serve as a universal *agr* antagonist by inhibiting *agr*-signaling and virulence factor expression by *agr*I–IV clinical isolates.

### ROS-dependent oxidation of LDL but not of AIP3 mediates antagonism of *agr*III-dependent quorum sensing

**Figure 4:**
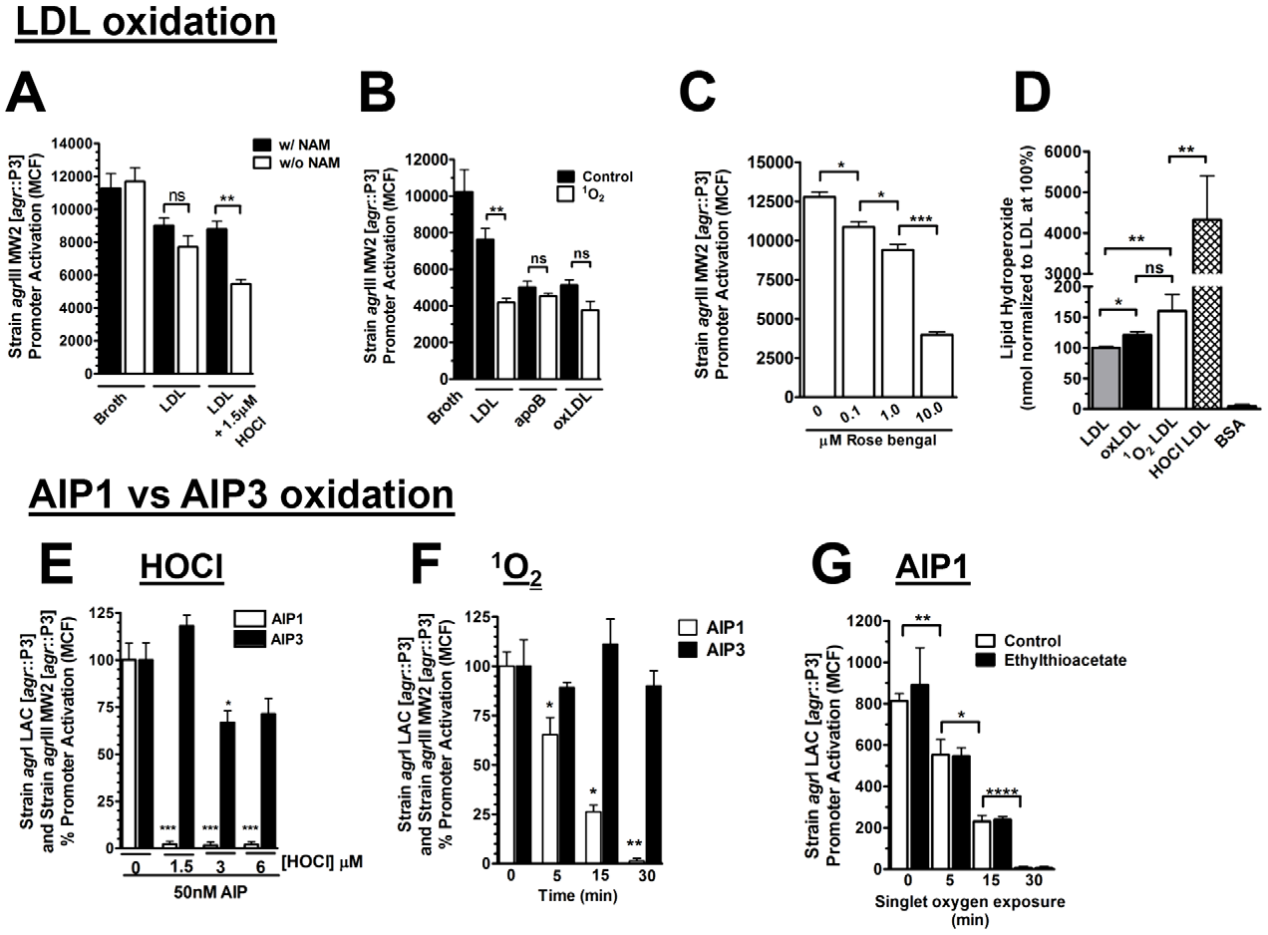
ROS-dependent oxidation of LDL but not of AIP3 mediates antagonism of *agr*III-dependent quorum sensing. (A, B) LDL, apoB or oxLDL at equimolar concentrations of apoB were treated as shown with (A) HOCl for 30 min at 37°C, with or without prior addition of the ROS scavenger N-acetylmethionine (NAM), or (B) singlet oxygen (5 min on ice). 10 nM ROS-treated lipoproteins or controls were cultured overnight with *agr*III MW2 [*agr*::P3-yfp] plus 50 nM AIP. *agr*:P3 promoter activation was measured by flow cytometry. (C) Strain *agr*III MW2 [*agr*::P3-yfp] was cultured overnight with 50 nM AIP3 plus LDL treated with increasing concentrations of singlet oxygen. (D) Measurement of LDL lipid oxidation before and after exposure to singlet oxygen or HOCl. Commercially available oxLDL and bovine serum albumin are given as controls. Data represent the mean ± SEM. (E) AIP1 and AIP3 were incubated with buffer or HOCl for 30 min at 37°C, treated with NAM to scavenge remaining ROS, and cultured at 50 nM for 2 h with *agr*I isolate LAC [*agr*::P3-yfp] or *agr*III MW2 [*agr*::P3-yfp], respectively. Data was measured by flow cytometry. Data points represent the mean ± SEM normalized to 100% activation by untreated AIP and statistics are in reference to this control. (F) AIP1 and AIP3 were exposed to singlet oxygen by incubation with rose bengal in the presence or absence of light, prior to culture with *agr*I isolate LAC [*agr*::P3-yfp] or *agr*III MW2 [*agr*::P3-yfp]. Data points represent the mean ± SEM normalized to 100% activation by untreated AIP and statistics are in reference to this control. (G) AIP1 was combined with buffer or a 10-fold molar excess of ethylthioacetate prior to exposure to singlet oxygen, followed by culture with *agr*I isolate LAC [*agr*::P3-yfp]. Data points represent the mean ± SEM of the mean channel fluorescence. ns, not significant; *, p<0.05; **, p<0.01; ***, p<0.001; ****, p<0.0001.

Based on published literature and our *in vivo* data [45], [46], [59]–[62], we postulated that *in vitro* oxidation of LDL by ROS would be sufficient to promote apoB-mediated antagonism of *agr*III-signaling. Previous *in vitro* studies have shown that isolated neutrophils induce lipid oxidation of LDL [45], [46], [63]. Therefore, we first determined the ability of HOCl, a ROS released by activated neutrophils via Nox2 and myeloperoxidase [57], [64], to enhance inhibition of *agr*::P3 promoter activation by LDL in the *agr*III isolate MW2. Exposure of LDL to HOCl significantly increased antagonism of AIP3-induced *agr*::P3 promoter activation compared to control LDL (Fig. 4A). This effect was dependent upon oxidative modification of LDL because the ROS scavenger N-acetylmethionine (NAM) blocked the increase in antagonism. To demonstrate that increased *agr*III-antagonism following oxidation of LDL is not limited to a single ROS, we evaluated singlet oxygen for the ability to enhance *agr*III antagonism by LDL. Singlet oxygen (^1^O_2_) is a strong oxidizing agent released by activated neutrophils and contributes significantly to neutrophil extracellular trap (NET) formation, ozone mediated bacterial killing and ozone formation in atherosclerotic plaques as well as conformational changes of apoB within LDL [38], [56], [65], [66]. In addition, ^1^O_2_ induces lipid oxidation of LDL [63], [67]. As expected, exposure of LDL to ^1^O_2_ (Fig. 4B) significantly increased the ability of LDL, but not apoB or oxLDL, to antagonize *agr*III-signaling and antagonism increased along with the oxidant dose (Fig. 4C). In addition, increased antagonism of *agr*III-signaling by ^1^O_2_ or HOCl-treated LDL corresponded with a significant increase in LDL lipid oxidation compared to control (Fig. 4D). Therefore, *in vitro* oxidation of LDL by ROS representative of activated Nox2 is sufficient to significantly increase apoB-mediated antagonism of *agr*III-dependent quorum sensing compared to untreated LDL.

Extravasation of activated neutrophils to sites of *agr*I *S. aureus* infection makes available extracellular ROS that provide defense in part by oxidizing the C-terminal methionine of AIP1 rendering it biologically inactive [27]. Because AIP3 lacks this methionine and the susceptibility of the common AIP thiolactone to oxidative inactivation has not been addressed, we postulated that AIP3 would be resistant to inactivation by ROS relative to AIP1. At concentrations of HOCl that readily inactivated AIP1, AIP3 retained biologic function (Figure 4E). At higher HOCl concentrations, there was a partial loss in AIP3 function that most likely resulted from fragmentation of the peptide as mass spectrometry failed to identify AIP3 species with charge to mass ratios suggestive of linearization or addition of oxygen atoms (data not shown). In contrast, mass spectrometry clearly revealed oxidation of the AIP1 methionine residue as was previously reported (data not shown) [27]. To further demonstrate the resistance of AIP3 to ROS-mediated inactivation, we assayed singlet oxygen for the ability to inactivate AIP3. As expected, AIP3 was resistant to inactivation by ^1^O_2_ under conditions in which AIP1 signaling decreased rapidly as a function of time (Fig. 4F). These results were not due to changes in bacterial growth resulting from exposure to residual ROS, as the cfus were consistent between both treated and untreated groups (data not shown). Because both AIP1 and AIP3 contain the 5-membered thiolactone ring, these results suggest that this ring is not a target for inactivation under the conditions examined here. To confirm that the AIP thiolactone is not a primary target for oxidative modification, we evaluated the ability of excess ethylthioacetate, a thiolactone mimetic, to protect AIP1 from oxidative loss of function by ^1^O_2_. At 10-fold molar excess, ethylthioacetate did not protect AIP1 from inactivation by ^1^O_2_ (Fig. 4G), indicating that the thiolactone does not compete with the AIP1 methionine for oxidative modification and therefore the thiolactone is not a primary target of oxidation. These data demonstrate that these ROS do not provide defense against *agr*III-mediated signaling by direct oxidative inactivation of AIP3 but rather through enhancement of apoB-mediated control of *agr*III-virulence by oxidative modification of LDL.

ROS-mediated inactivation of AIP1 but not AIP3 suggests that methionine is the primary target for oxidative inactivation of AIP. To extend these observations to the role of ROS in oxidative inactivation of other AIPs, we postulated that ^1^O_2_ would inactivate AIP4, which includes a C-terminal methionine residue, but that AIP2 which lacks methionine would be resistant to inactivation. As predicted, exposure of AIP4 to ^1^O_2_ significantly inhibited AIP4-induced *agr*::P3 promoter activation in the *agr*IV isolate MN TG, whereas AIP2 function remained unchanged (Fig. S4). These data suggest that in addition to oxidation of LDL, ROS can play an independent role in defense against *agr*I and *agr*IV quorum sensing through direct oxidative inactivation of AIP1 and AIP4.

### Exogenous oxLDL restores *in vivo* antagonism of *agr*III-signaling

**Figure 5:**
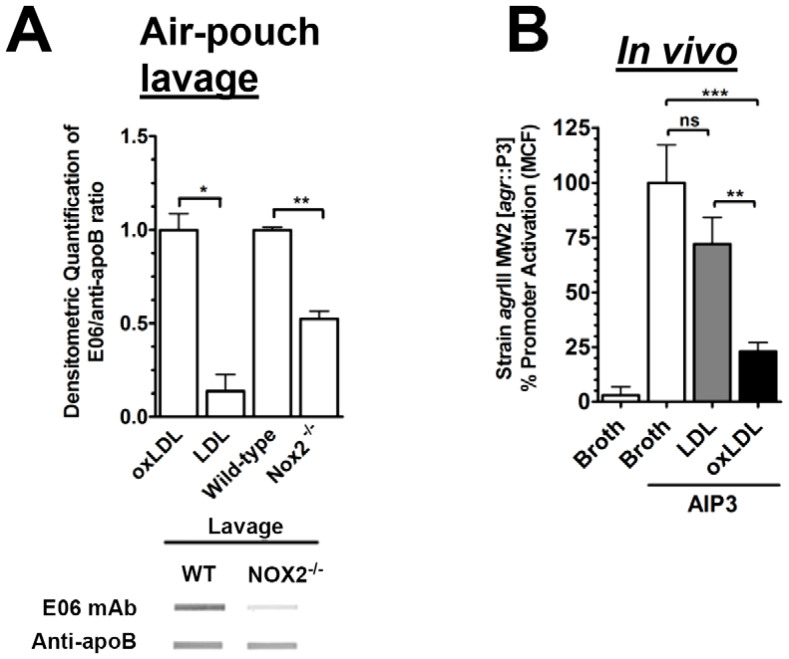
Exogenous oxLDL restores in vivo antagonism of *agr*III-signaling. (A) Control LDL and oxLDL plus air-pouch lavage from MW2 infected wild-type and *Nox2^−/−^* mice was vacuum transferred to nitrocellulose and stained for oxLDL using monoclonal antibody E06 or rabbit polyclonal antibody to apoB. A representative blot is shown. Band intensity was quantified using Carestream Molecular Imaging software (New Haven, Connecticut), and data normalized to oxLDL or wild-type lavage with E06/apoB ratios equal to 1. (B) Air-pouches were generated on the backs of 8 to 12 week old *Nox2^−/−^* mice treated with 4APP as previously described. At time zero, 4×10^7^ cfu of early exponential phase *agr*III isolate MW2 [*agr*::P3-yfp] were injected into the air-pouch along with saline control or the following as indicated: 100 nM AIP3 and either buffer control, 100 nM LDL or 100 nM oxLDL. After 4 h pouches were lavaged and *agr*::P3 promoter activation assessed by flow cytometry. Data reported are the mean ± SEM normalized to PBS/AIP3 control. ns, not significant; *, p<0.05; **, p<0.01; ***, p<0.001.

Serum proteins extravasate into inflamed tissues and aid in host defense by providing antibody and complement necessary for opsonization [68]. We predicted that extravasated oxLDL present at the site of infection would also contribute to host defense by antagonizing *agr*III invasive infection *in vivo*. To address this, we first determined relative levels of oxLDL and LDL in lavages of air-pouches of wild-type and *Nox2^−/−^* mice 28 hours after infection with MW2. As expected, oxLDL was present in lavages from wild-type mice who were protected from *agr*III invasion, but was largely absent in lavages from *Nox2^−/−^* mice which were highly susceptible to MW2 invasion (Fig. 5A). From these results, we predicted that the addition of oxLDL, but not native LDL, to the air-pouch at the time of infection of *Nox2^−/−^* mice would inhibit *agr*III-signaling. To test this we infected air-pouches of 4APP-treated *Nox2^−/−^* mice with *agr*III isolate MW2 [*agr*::P3-yfp] in the presence of oxLDL, LDL or buffer control. Oxidized LDL, but not LDL, introduced into the air-pouch at the time of infection significantly antagonized AIP3-mediated *agr*::P3 activation in the resulting air-pouch lavage four hours after infection (Fig. 5B). Weight loss is a primary measure of morbidity in this model and mice treated with oxLDL at the time of infection lost significantly less of their total body weight at four and eight hours post-infection compared to controls (Fig. S5). Thus the presence of oxLDL in the air-pouch at the time of infection significantly inhibited both *agr*-signaling and morbidity as measured by weight loss. Both the presence of oxLDL in air-pouch lavages from infected wild-type mice and restoration of *agr*III antagonism by addition of oxLDL, but not LDL, to the air-pouch of 4APP-treated *Nox2^−/−^* mice, indicates that Nox2-mediated apoB control of *agr*III-dependent signaling works directly at the site of infection to prevent *agr* type III-dependent signaling and its pathological consequences.

## Discussion

Host innate immunity is critical for maintaining a defensive barrier against opportunistic pathogens such as *Staphylococcus aureus* which colonize skin and mucosal surfaces. In order to breach these defensive barriers, *S. aureus* uses the *agr* quorum sensing system to coordinate expression of virulence genes needed for invasion and to evade host defensive mechanisms. The fact that most humans are able to limit *S. aureus* infections to minor ones of skin and skin structures [69] suggests that host factors capable of inhibiting quorum sensing signaling mediated by each of the four *agr* alleles could contribute to host defense against invasive infection. This antagonism would thus promote host bacterial killing and clearing mechanisms. Here, we extend our knowledge of host inhibition of *S. aureus agr*I-signaling to host control of quorum-sensing dependent virulence mediated by *agr*III. Importantly, our studies into host control of *agr*III signaling revealed an important role for the conformation of apoB within lipoprotein particles such that oxLDL was found to be a universal inhibitor of *agr* signaling in all four *agr* alleles. We previously reported that both apolipoprotein B, the structural protein of VLDL and LDL, and ROS generated by the phagocyte NADPH oxidase (Nox2), provide unique barriers against *S. aureus agr*I-mediated virulence and the loss of either significantly increases susceptibility to invasive infection [24], [27]. Specifically, Nox2 derived ROS directly inactivate AIP1 by methionine oxidation whereas the apoB component of LDL binds and sequesters AIP1 to prevent *agr*I-mediated virulence. This current work demonstrates that the roles of apoB and Nox2 in defense against *agr*III-mediated infections are interdependent. Unlike AIP1, AIP3 is resistant to oxidation and direct inactivation by ROS characteristically produced by Nox2. Instead, the contribution of Nox2 to host innate defense against *S. aureus agr*III-dependent quorum sensing is through oxidation of LDL. ApoB in the context of oxLDL, and not LDL, provides optimal host defense against *S. aureus agr*III infection by binding the secreted signaling peptide, AIP3, and preventing expression of the *agr*-driven virulence factors which mediate invasive infection. Our results suggest that oxidation of LDL facilitates a conformational change in apoB to one required for optimal binding of AIP3 and apoB-mediated defense against *agr*III-dependent virulence. Intriguingly, *agr*I-, *agr*II- and *agr*IV-signaling was also antagonized by oxLDL *in vitro*, suggesting that apoB within oxLDL is sufficient for antagonism of signaling by each of the four *agr* alleles. Each of the four *S. aureus agr* alleles are associated with invasive human infections which may range in severity from mild skin and soft tissue infections to severe disease such as osteomyelitis, endocarditis, necrotizing pneumonia and bacteremia [31]. Our data suggest that oxLDL may provide protection against *S. aureus* infections of any *agr* type in which *agr*-signaling contributes to pathogenesis. In contrast, we would predict that oxLDL would play a minimal role in host defense in chronic or device related infections in which *agr* signaling may be less relevant [70]–[73].

Our data show that LDL and oxLDL are present at the site of infection and prevent quorum-sensing dependent invasion of *agr*III *S. aureus* into the underlying dermis. This suggests that other serum proteins which extravasate to the site of infection may also contribute to defense against *agr*-mediated signaling. Recently, Surewaard *et al* [74] described a novel role for VLDL, LDL and HDL in binding and inactivation of phenol soluble modulins (PSM), an important group of *agr*-regulated *S. aureus* virulence factors. Among these lipoproteins, HDL was attributed with the highest PSM scavenging activity and, using a wide variety of *in vitro* assays, demonstrated inhibition of all PSMs investigated. However, unlike our data demonstrating apoB binding and antagonism of AIP1-4, lipoprotein-mediated inhibition of PSMs was attributed to the lipid and not the protein components of the lipoprotein particles. Another serum component, hemoglobin, also suppresses *agr* function when released from red blood cells [26], [75]. Once *agr*-signaling is initiated and PSMs as well as hemolysins needed to lyse RBCs for release of hemoglobin have begun to be expressed, both lipoprotein-mediated scavenging of PSMs and hemoglobin-mediated suppression of *agr*-signaling could clearly contribute to prevention of further *agr*-mediated virulence and invasion of host tissues. Although these additional mechanisms of controlling quorum-sensing dependent *S. aureus* virulence may contribute in part to our *in vivo* results, the following factors support the primary role of apoB-mediated control of *agr*-dependent virulence in our model: 1) apoB binds immobilized AIP1-4 and apoB and oxLDL antagonize *agr*-dependent P3 promoter activation, RNAIII, *psmα* and *hla* transcription and Hla production *in vitro* in both laboratory strains and clinical isolates, 2) addition of oxLDL, but not LDL, to MW2 infected air-pouches of LDL-deficient *Nox2^−/−^* mice was sufficient to suppress quorum-sensing independent of other lipoproteins and 3) compared to untreated mice, 4APP-treated wild-type mice with equivalent oxLDL levels were not more susceptible to MW2 invasive infection, suggesting that oxLDL-mediated control of *agr*III-dependent virulence factor expression predominated and offset the need for lipoprotein scavenging of PSMs. Therefore, apoB plays a unique role in inhibition of *agr* signaling upon initiation of infection. All of these findings highlight the host's multi-tiered innate defense system to combat *S. aureus agr*-mediated virulence and suggest that conditions which result in decreased lipoprotein levels may contribute on more than one level to host susceptibility to *S. aureus* invasive infection.

Our data comparing oxLDL and LDL binding of AIP1 versus AIP3 demonstrate that whereas oxLDL provides optimal binding and antagonism of AIP3, both lipoprotein particles are equally efficient at binding AIP1. Such differences in the ability of apoB-containing lipoprotein particles to bind and antagonize these AIPs may result from multiple factors. One explanation could be that although both peptides contain the conserved thiolactone bond, the length and sequence variation between AIP1 and AIP3 may suggest that each has a unique binding site on apoB. Because oxidation of LDL is known to alter the conformation of apoB within the particle, oxLDL may present apoB in the ideal conformation for binding AIP3 [39], [40], [76], whereas the binding site for AIP1 may be located in a region of apoB not subject to conformational change. The importance of apoB conformation in antagonizing *agr*III-virulence is supported by the ability of an apoB-specific antibody to block AIP3 binding by both apoB and oxLDL, but not LDL. Further investigations using antibody blocking and competition binding studies should help to elucidate differences in binding of AIP 1-4 by the different apoB-containing lipoprotein particles and to determine whether there is a single site or multiple sites for AIP binding to apoB. At present however, the specific binding site(s) for AIP 1-4 within the three-dimensional structure of this 515 kDa protein and the relevant binding affinities require further investigation.

Nox2 activation contributes to phagocyte oxidative responses and in this regard patients with severe sepsis have significantly elevated levels of serum oxLDL suggesting that Nox2 can contribute to oxLDL formation during infection [64], [77]–[79]. Interestingly, we observed increased oxLDL in the serum of wild-type C57BL/6 mice compared to serum from *Nox2^−/−^* mice prior to infection indicating that Nox2-derived ROS contribute to constitutive oxidation of LDL in the circulation. This may be explained in part by expression of Nox2 in other cell types including endothelial cells and adventitial fibroblasts, where it participates in cell cycle regulation and apoptosis [80]. For example, in adventitial fibroblasts, Nox2-derived ROS act in an autocrine and paracrine fashion to mediate angiogenesis and vessel homeostasis [81], and could contribute to the pre-infection levels of circulating oxLDL seen in wild-type mice. In addition, Kupffer cells within the liver, which are among the first cells to sense endotoxin coming from the gut, could contribute to oxidation of LDL by constitutive release of Nox2-derived ROS in response to endotoxin released by gut microbiota [82], [83]. Epidermal Langerhans cells might also contribute to constitutive ROS production as they survey the skin for microbes [84]–[86]. Therefore, Nox2 activity in these other cell types may contribute to a basal level of circulating oxLDL, which in turn may play a sentinel role in innate immune defense against both *agr*I and *agr*III-mediated *S. aureus* invasive infection.

Additional roles for both ROS and apoB in innate defense against *S. aureus* have recently been reported. First, Sun and colleagues demonstrated that oxidation of amino acid Cys199 of AgrA disrupted DNA binding and inhibited *agr*I-dependent expression of RNAIII and psmα *in vitro* [87]. This mechanism of oxidation sensing by the *agr* system is viewed as a bacterial mechanism to counter oxidative stress. However, oxidation of the AgrA disulfide redox switch may also be considered a form of host defense against *S. aureus agr*-signaling. This mechanism is distinct from ROS-mediated inactivation of AIP or oxidation of LDL as oxidation of either AgrA, AIP1 or LDL in isolation is sufficient to antagonize *agr*-signaling. Second, apoB and LDL were shown to inhibit LTA-induced cytokine release from immune cells through direct interaction with LTA [88]. Thus, apoB could modulate the host response to *S. aureus* independent of its role in antagonizing *agr*-signaling. If this were the primary function of apoB and oxLDL in our current work we would have expected to see a significant role for reduced serum apoB in susceptibility to an *agr* negative infection in which LTA would be equivalent and we did not. Together, these reports demonstrate multiple distinct and independent roles for both ROS and apoB in both *agr*-dependent and *agr*-independent host defense against *S. aureus* infection.

Although to our knowledge this is the first description of oxLDL as an innate defense factor controlling quorum-sensing dependent virulence, oxLDL contributes in other direct and indirect ways to host defense. For example, oxLDL inhibits hepatitis C virus cellular entry *in vitro* [89] and reduces infection by the malaria parasite *Plasmodium falciparum* by binding the scavenger receptor SR-BI which is also utilized by the parasite [90]. OxLDL may also indirectly contribute to host antimicrobial defense. Autoantibodies generated against lipid components of oxLDL have demonstrated protection against infection by some strains of *Streptococcus pneumoniae* and *Haemophilus influenzae* [91]. However, other bacteria-oxLDL interactions are harmful to the host. For example, certain strains of *Helicobacter pylori* enhance atherosclerosis by increasing serum oxLDL [92] and the mitogenic activity of *Chlamydia pneumonia* in vascular smooth muscle cells is enhanced by oxLDL [93]. Although our understanding of the myriad contributions of oxLDL to host antimicrobial defense or microbial pathogenesis is clearly limited, the identification of oxLDL as a barrier against *S. aureus agr* dependent quorum-sensing by each of the four *agr* alleles provides new insight into the role of oxLDL in host defense.

## Materials and Methods

### Ethics statement

Animal work in this study was carried out in strict accordance with the recommendations in the Guide for the Care and Use of Laboratory Animals of the National Institutes of Health, the Animal Welfare Act and US federal law. The protocol was approved by the Institutional Animal Care and Use Committee (IACUC) of the Research Service of the New Mexico VA Health Care Service.

### Reagents

Synthetic AIPs were synthesized by Biopeptide Co., Inc (San Diego, CA) as described [94] and stored in DMSO at ^−^80°C. Purified human LDL and oxLDL (Biomedical Technologies Inc., Stoughton, MA and Kalen Biomedical, Montgomery Village, MD) and purified apoB (US Biological, Swampscott, MA) were assayed for apoB content using a commercially available kit (ALerCHEK, Inc., Springvale, Maine). The following reagents were obtained as follows: tributyrin, 4-aminopyrazolo-(3,4-D) pyrimidine (4APP), HOCl, Rose Bengal, N-acetymethionine (NAM), ethylthioacetate, (Sigma-Aldrich, St. Louis, MO); E06 monoclonal Ab (Avanti Polar Lipids, Alabaster, AL); Rabbit anti-apoB (Abcam, Cambridge, MA); Goat anti-apoB (Santa Cruz Biotechnology, Santa Cruz, CA); Goat IgG (R&D Systems, Minneapolis, MN).

### Bacterial strains and cultures

The bacterial strains used in this study were as follows: AH1677 (strain *agr* I LAC [*agr*:P3-yfp]), AH1747 (strain *agr* III MW2 [*agr*:P3-yfp]), AH430 (strain *agr*II 502a [*agr*:P3-yfp]) and AH1872 (strain *agr*IV MN TG [*agr*:P3-yfp]) were provided by Dr. Alex Horswill (University of Iowa) [41]; USA300 strain LAC and USA400 strain MW2 and their *agr* deletion mutants were provided by Dr. Mike Otto. *agr*IV clinical isolates (NRS165 and NRS166) were obtained through the Network on Antimicrobial Resistance in *Staphylococcus aureus* (NARSA) program supported under NIAID, NIH Contract No. HHSN272200700055C. Additional clinical MRSA and MSSA isolates were provided by Dr. Larry Massey, New Mexico VAHCS. To generate synchronized early exponential phase, non-fluorescent bacteria, frozen stocks were cultured in Trypticase soy broth (TSB) (Becton Dickinson, Franklin Lakes, NJ) as described [28]. CFU were determined after washing and sonication to disrupt clumps by plating serial dilutions on blood agar (Becton Dickinson, Franklin Lakes, NJ).

### RNAIII promoter activation assays

Early exponential phase, nonfluorescent reporter bacteria (2×10^7^/ml) were incubated in TSB in polystyrene tubes with shaking (200 rpm, 37°C) for the indicated times with either broth, synthetic AIP, AIP treated with ROS, or antagonists including lipoprotein particles or apoB. After incubation, bacteria were washed by centrifugation at 3000 rpm for 4 min at 4°C in PBS with 0.1% Triton X-100, sonicated, cultured for CFU where indicated, and then fixed with 1% paraformaldehyde containing 25 mM CaCl2 for analysis by flow cytometry (Accuri C6, BD Accuri Cytometers, Ann Arbor, MI). Promoter activation was demonstrated as fluorescence induction and measured as the mean channel fluorescence (MCF) of YFP-positive bacteria.

### Quantitative RT-PCR

Quantitative RT-PCR was carried out as previously described [24]. Early exponential phase isolates were cultured as described and as indicated in the figure legends. RNA was isolated and purified using the Qiagen RNAprotect Bacteria Reagent and RNeasy Mini Kit (Qiagen, Valencia, CA). cDNA was generated using a high capacity cDNA RT kit with an RNAse inhibitor (Applied Biosystems, Foster City, CA) and an Eppendorf Mastercycle thermocycler (Hamburg, Germany). RNAIII was quantified relative to 16S RNA using a probe based assay as described with minor modifications [95]. Briefly, cDNA was quantified using an ABI7300 Real-Time PCR system with Taqman Gene Expression master mix, ROX probe/quencher, and appropriate primer sequences (Applied Biosystems). Each experiment was performed in duplicate and samples assayed in triplicate. The primer-probe sequences used were as follows: *psm*α forward primer 5′-TATCAAAAGCTTAATCGAACAATTC-3′, *psm*α probe 5′-6-FAM-AAAGACCTCCTTTGTTTGTTATGAAATCTTATTTACCAG-BHQ-2-3′, *psm*α reverse primer 5′- CCCCTTCAAATAAGATGTTCATATC-3′, *hla* forward primer 5′-ACAATTTTAGAGAGCCCAACTGAT-3′, *hla* probe 5′-6-FAM-AAAAAGTAGGCTGGAAAGTGATA-BHQ-2-3′, *hla* reverse primer 5′-TCCCCAATTTTGATTCACCAT-3′, *RNAIII* forward primer 5′-AATTAGCAAGTGAGTAACATTTGCTAGT-3′, *RNAIII* probe 5′-6-FAM-AGTTAGTTTCCTTGGACTCAGT-GCTATGTATTTTTCTT-BHQ-2-3′, *RNAIII* reverse primer 5′-GATGTTGTTTACGATAGCTTACATGC-3′, *16S* forward primer 5′- TGATCCTGGCTCAGGATGA-3′, *16S* probe 5′-6-FAM-CGCTGGCGGCGTGCCTA-BHQ-2-3′, *16S* reverse primer 5′-TTCGCTCGACTTGCATGTA-3′.

### Virulence factor assays

Lipase and alpha hemolysin activity was measured using 0.45 µm filtered cultured supernatants from bacterial strains grown overnight as described above in 5 ml TSB with and without 50 nM LDL or oxLDL. Addition of LDL or oxLDL to supernatants from untreated cultures was used as additional controls. Lipase was measured as described using a triglyceride substrate, tributyrin [96]. Alpha hemolysin was measured as described using rabbit erythrocyte lysis [97]. One unit of hemolytic activity was defined as the amount of bacterial supernatant able to liberate half of the total hemoglobin from the erythrocytes.

### *agr* typing

*agr* typing of clinical isolates was performed using PCR as previously described with minor modifications [98]. In brief, cell pellets from overnight bacterial cultures were suspended in Tris-EDTA buffer (10 mM Tris, 1 mM EDTA), added to 0.1 mm Zirconia beads (Biospec) and lysed using a bead beater (Biospec). Following centrifugation, the liquid phase was diluted 50-fold and specific DNA quantified by real-time PCR using an ABI7300 Real-Time PCR system with Taqman Gene Expression master mix, along with *agr* type specific primers and probes [98]. Each assay included positive control DNA from *agr* group I isolate LAC, *agr* group II isolate 502A and *agr* group III isolate MW2.

### Oxidant treatment of AIP and lipoproteins

Oxidation of AIP or LDL was performed using HOCl or singlet oxygen. HOCl (Sigma) was diluted and incubated with AIP or antagonist (lipoprotein or apoprotein) at 37°C for 30 min. The HOCl concentration was determined by its absorbance at 292 nm (pH 12, ε_292_ = 350 M^−1^•cm^−^1) [27]. Reactions were carried out in sterile microfuge tubes in 100 µl volumes and contained 1 µM AIP or 2 µM lipoprotein based on apoB concentration. Residual ROS were scavenged by the addition of 10 mM NAM following HOCl treatment, before addition to the bacterial reporter assay. Singlet oxygen was generated using 10 µM Rose Bengal with or without exposure to 150 W light for 5 min unless otherwise noted [99], [100]. To control for heat induction, light was applied through a cold water filled 9.5 cm filter positioned directly above the samples. Sample volumes and concentrations were identical to those used for the HOCl oxidation assays. A tenfold molar excess of ethylthioacetate relative to AIP1 was used for analysis of thiolactone susceptibility to oxidation. LDL oxidation was assayed using either the LPO Assay kit or by determination of 8-isoprostane levels [101], [102] using the 8-Isoprostane EIA Kit and 9-Isoprostane Affinity Purification kit (Cayman Chemical Co, Ann Arbor, MI) as per the manufacturer's instructions. For determination of 8-isoprostane levels, butylhydroxytoluene was added to LDL samples to 0.005% and samples were flash frozen and stored at −80°C until analysis.

### Immunoassay

Immunblot analyses were performed to detect apoB and oxLDL in mouse serum and air-pouch lavages. Samples were loaded onto nitrocellulose membranes, presoaked in Tris Buffered Saline (TBS), using a microfiltration apparatus (BioRad, Hercules, CA). Slots were washed with additional TBS following sample application. Membranes were blocked for 30 min using SuperBlock (Pierce, Rockford, IL), then probed with SuperBlock containing rabbit anti-apoB or E06 anti-oxLDL for 30 min at room temperature (RT). Unbound primary antibody was removed by washing 3× with TBS, 0.1% Tween20 (TBST), followed by incubation with the appropriate alkaline phosphatase (AP) conjugated secondary antibody also in SuperBlock. After 30 min at 25°C. RT, blots were washed 3× with TBST and 1× with TBS. Blots were developed using nitro-blue tetrazolium and 5-bromo-4-chloro-3′-indolyphosphate (NBT/BCIP, Pierce). Band intensity was quantified using Carestream Molecular Imaging software (Rochester, NY, USA).

### Surface plasmon resonance

Surface plasmon resonance was performed using a Biacore ×100 (Biacore Life Sciences, GE Healthcare) to analyze the interaction of the analytes (LDL, apoB and oxLDL) with immobilized ligand (AIP) as previously described [24]. N-terminally biotinylated AIP in both native and linear conformations was immobilized on streptavidin sensor chips (GE Healthcare, Piscataway, NJ) according to the manufacturer's protocol. The chips were regenerated with 50 mM NaOH, 1 M NaCl and then washed with immobilization buffer (10 mM Hepes, pH 7.4, 150 mM NaCl, 3 mM EDTA). Biotinylated AIPs were pulsed onto the chip at a concentration of 1 µM for 420 s at a flow rate of 10 µl/min followed by extensive washing. For binding studies, analytes were applied at 10 nM in running buffer (10 mM Hepes, pH 7.4, 250 mM NaCl, 3 mM EDTA) at a flow rate of 10 µl/min with a contact time of 60 s and a dissociation time of 60 s. Chip platforms were regenerated using a 60 s wash with 0.5% SDS followed by 120 s stabilization period. For each experiment specific binding was measured as the RU generated by analyte binding to the test surface minus RU generated by analyte binding to the reference surface (streptavidin without biotin-AIP). The Biacore evaluation software (×100 Version 1.0) was used to analyze the results. All analyses were performed at 25°C.

### Mice

C57BL/6 mice (8–12 wk, ≈22–28 g) from Charles Rivers (Wilmington, MA), and *Nox2* knockout mice on the C57BL/6 background (*Nox2^−/−^*) [42] from Jackson Laboratory (Bar Harbor, ME) were gender- and age-matched. Mice receiving 4APP treatment were injected i.p. 48 hours and 24 hours prior to infection with 100 µl of 5 mg/ml 4APP prepared by dissolving in 1 M HCl at 100 mg/ml and diluted to 5 mg/ml in 0.025 M phosphate buffer (pH 8). The solution was adjusted to pH 4 with 7.5% NaHCO3 immediately before injection. Total cholesterol levels were determined using a kit (Thermo Electron, Louisville, CO) per manufacturer's instructions. 4APP treated mice typically showed a 40–50% reduction in serum cholesterol levels compared to wild-type mice. Cholesterol levels specific to VLDL/LDL were determined using a kit (BioVision, Mountain view, CA) according to the manufacturer's protocol.

### Air pouch infection model

Subcutaneous air pouches were created and infected with early exponential phase bacteria at the indicated dose as previously described [27], [28]. Twenty-eight hours post infection, the mice were scored for morbidity by the following scale: appearance: 0–4; natural behavior: 0–3; hydration status (skin pinch test): 0–3; provoked behavior: 0–4. The morbidity score is the sum of the scores in the 4 categories with a maximum of 14 at which point the mice were considered moribund. In addition, weight loss was measured and pouch, lavage and spleen CFU were determined as described [24], [27].

### Tissue invasion assay

Basolateral pouch tissue was excised, rinsed in PBS and fixed in 3% paraformaldehye containing 0.1 mM CaCl2 and 0.1 mM MgCl in PBS. Pouch tissue was then rinsed with PBS and stored in 25% sucrose until freezing. Pouches were frozen in O.C.T. compound using liquid nitrogen. Cryo blocks were stored at −80°C until sectioned. Cryosections (10 µm) were cut onto slides, fixed and permeabilized with acetone at −20°C for 5 minutes, rehydrated with PBS, and blocked overnight with 0.5% BSA, 5% normal rabbit serum, 0.1% Triton-X-100 in PBS. Slides were incubated for 1 h at 4°C with anti-*S. aureus* antibody (GeneTex, San Antonio, TX) conjugated with Alexafluor 488 (Molecular Probes/Invitrogen). Slides were rinsed and mounted in Prolong Gold Antifade (Molecular Probes/Invitrogen). Images were acquired on a Zeiss LSM 510 confocal microscope. Bacterial density as a measure of tissue invasion into the dermis was quantified from LSM images using Slidebook software (Intelligent Imaging Innovations, Denver, CO). Regions of interest were selected using histiologic criteria for the dermis (3000 µm^2^) and the total area and the portion of that area stained for *S. aureus* quantified in microns^2^. Values are displayed as percentage of square microns of staining within the identified area. A minimum of 20 sections were examined for each experimental condition.

### FPLC isolation of mouse LDL

LDL was isolated from mouse serum basically as previously described [103], [104]. Specifically, protease inhibitors and antioxidants were immediately added to freshly prepared serum (Roche Complete Protease Inhibitor Cocktail Tablets, 1 mM EDTA, 1 mM DTT and 20 mM L-ascorbate), and the serum and lipoprotein fraction were stored under an argon atmosphere. LDL was separated using gel filtration chromatography at 4°C with a GE Life Sciences ÄTKA FPLC system and two Superose 6 10/300 GL columns in series, using 20 mM HEPES, 250 mM NaCl, 1 mM EDTA buffer at a flow rate of 0.3 mL min-1. Up to 600 µL serum was loaded, 1 mL fractions collected, and the eluent was monitored by the absorbance at 280 nm. The LDL containing fractions were supplemented with protease inhibitors and antioxidants, pooled and concentrated with Amicon Ultra centrifugal filter (100 kDa MWCO). Immediately before use, LDL was buffer exchanged and concentrated into PBS using centrifugal filters. Total protein concentration was determined using A_280_ = 1 mg/ml.

### Statistical analyses

Data are displayed as the mean ± SEM. *In vitro* data were analyzed by the Student's t test and the *in vivo* results by the Mann-Whitney U test for nonparametrics.

## Supporting Information

Figure S1 **OxLDL antagonizes AIP binding and** ***agr*** **::P3 promoter activation mediated by** ***agr*** **I,** ***agr*** **II and** ***agr*** **IV alleles.** (A) Quantification of *psm*α transcript relative to 16S RNA produced by LAC or MW2 cultured 1 h in the presence of 50 nM AIP, plus 50 nM LDL or apoB. Data points represent the mean ± SEM normalized to *psm*α induction by AIP plus broth alone. Statistics are in reference to AIP plus broth. (B) *agr*I isolate LAC [*agr*::P3-yfp] or *agr*III MW2 [*agr*::P3-yfp] was cultured for 2 h with 50 nM AIP plus 10 nM LDL or oxLDL. Data points represent the mean ± SEM normalized to broth control. (C) LDL, apoB or oxLDL binding to immobilized AIP1 was measured by SPR. Data were normalized to the mean ± SEM of LDL binding. (D) Schematic representation of AIP2 and AIP4. (E) OxLDL binding to immobilized AIP2 and AIP4 was measured by SPR. (F) OxLDL antagonizes *agr*II and *agr*IV P3 promoter activation. AH430 (*agr*II) and AH1874 (*agr*IV) were cultured overnight with broth control or 10 nM oxLDL and *agr*::P3 promoter activation measured by flow cytometry. ns, not significant; *, p<0.05; **, p<0.01; ***, p<0.001.
(TIF)

Figure S2 **Effect of Nox2 deletion on oxidation of LDL and of 4APP on serum cholesterol associated with apoB-containing lipoprotein particles.** (A) Lipid oxidation of LDL purified from the serum of *Nox2^−/−^* mice relative to LDL from wild-type mice. (B) Serum was collected from wild-type mice treated with vehicle control or 4APP and the cholesterol content of the LDL/VLDL fraction determined. (C) Lipid oxidation of LDL purified from the serum of 4APP-treated wild-type mice relative to LDL from control treated mice. Data reported as the mean ± SEM. *, p<0.05; **, p<0.01.
(TIF)

Figure S3 **OxLDL antagonizes RNAIII transcription and** ***agr*** **-dependent virulence factor transcription and expression by MRSA and MSSA** ***agr*** **I, II and IV clinical isolates.** (A) **A**ddition of LDL or oxLDL to (A) purified Hla or (B) supernatant containing lipase has no direct impact on the activity of either virulence factor as measured by functional assay. (C–E) Clinical isolates were cultured 2 h with 50 nM AIP (*agr*I and II) or overnight without exogenous AIP (*agr*IV) plus 10 nM oxLDL or media control. Transcription of *RNAIII* and *hla* relative to *16S* rRNA was measured by qRT-PCR and Hla expression was assessed by functional assay and reported as percent activity relative to media control. *, p<0.05; **, p<0.01; ***, p<0.001; ****, p<0.0001.
(TIF)

Figure S4 **Singlet oxygen mediates oxidative inactivation of AIP4 but not AIP2.** AIP2 and AIP4 were exposed to singlet oxygen by incubation with rose bengal in the presence or absence of light, prior to culture with (A) AH430 or (B) AH1872, respectively. *agr*::P3 promoter activation was measured by flow cytometry. Data points represent the mean ± SEM of the mean channel fluorescence. ****, p<0.0001.
(TIF)

Figure S5 **Exogenous oxLDL reduces morbidity in** ***S. aureus agr*** **III infected 4APP-treated** ***Nox2^−/−^*** **mice at 4 and 8 h post-infection.** Air-pouches were generated on the backs of 8 to 12 week old *Nox2^−/−^* mice treated with 4APP as previously described. At time zero, 4×10^7^ cfu of early exponential phase *agr*III MW2 [*agr*::P3-yfp] were injected into the air-pouch along with 100 nM AIP3 and either 100 nM oxLDL or saline control. Weight loss was measured at the time of infection and at (A) 4 h and (B) 8 h post-infection and is shown as percent body weight lost relative to time zero. *, p<0.05; **, p<0.01.
(TIF)
