# Supplementary figures and images for: Nox2 Modification of LDL Is Essential for Optimal Apolipoprotein B-mediated Control of agr Type III Staphylococcus aureus Quorum-sensing
Source: PLoS Pathog. 2013 Feb 14;9(2):e1003166. doi: 10.1371/journal.ppat.1003166 (PMC3573103; doi:10.1371/journal.ppat.1003166)

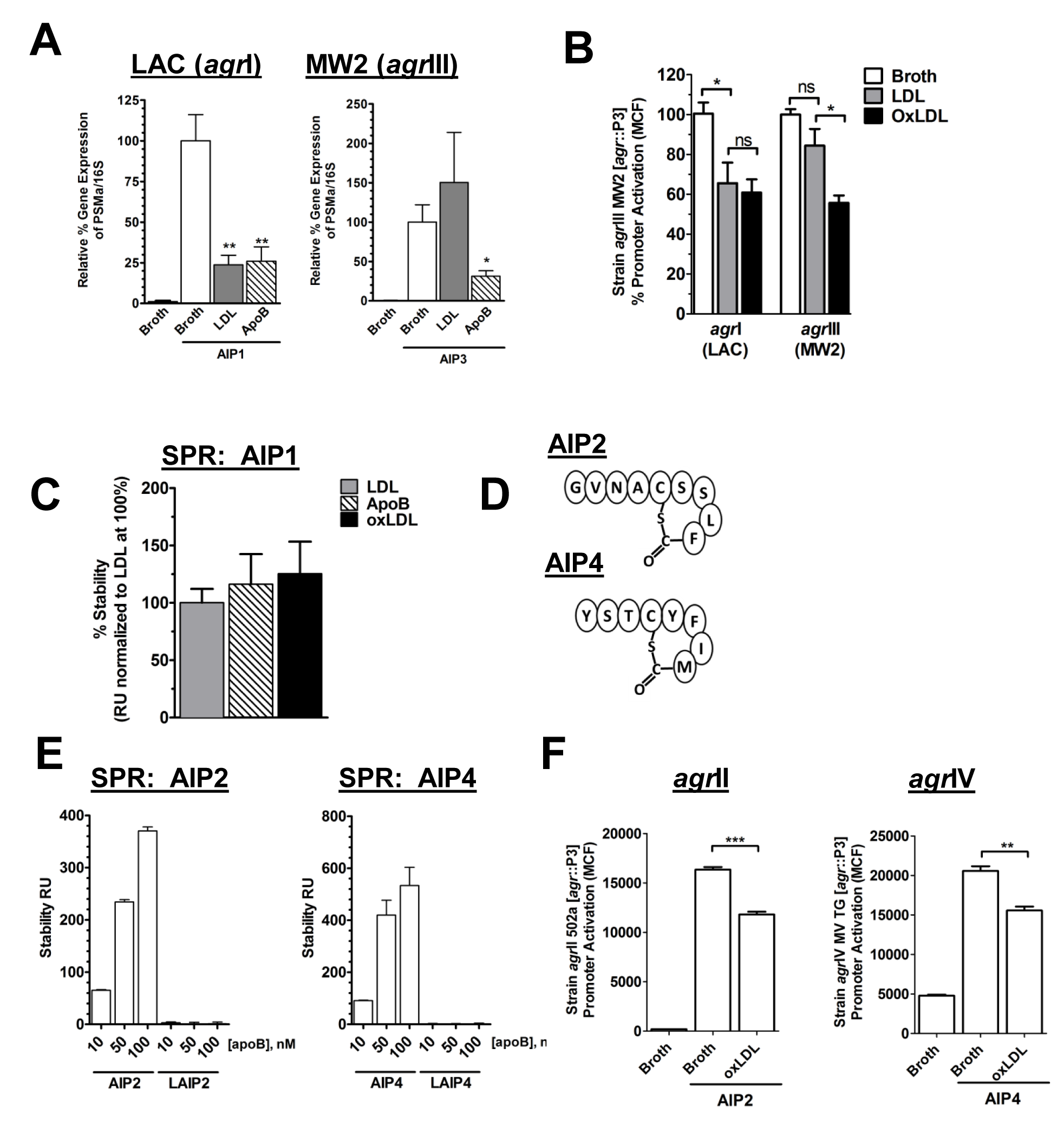

Supplement: Figure S1 — OxLDL antagonizes AIP binding and agr ::P3 promoter activation mediated by agr I, agr II and agr IV alleles. (A) Quantification of psmα transcript relative to 16S RNA produced by LAC or MW2 cultured 1 h in the presence of 50 nM AIP, plus 50 nM LDL or apoB. Data points represent the mean ± SEM normalized to psmα induction by AIP plus broth alone. Statistics are in reference to AIP plus broth. (B) agrI isolate LAC [agr::P3-yfp] or agrIII MW2 [agr::P3-yfp] was cultured for 2 h with 50 nM AIP plus 10 nM LDL or oxLDL. Data points represent the mean ± SEM normalized to broth control. (C) LDL, apoB or oxLDL binding to immobilized AIP1 was measured by SPR. Data were normalized to the mean ± SEM of LDL binding. (D) Schematic representation of AIP2 and AIP4. (E) OxLDL binding to immobilized AIP2 and AIP4 was measured by SPR. (F) OxLDL antagonizes agrII and agrIV P3 promoter activation. AH430 (agrII) and AH1874 (agrIV) were cultured overnight with broth control or 10 nM oxLDL and agr::P3 promoter activation measured by flow cytometry. ns, not significant; *, p<0.05; **, p<0.01; ***, p<0.001. (TIF) [file ppat.1003166.s001.tif]

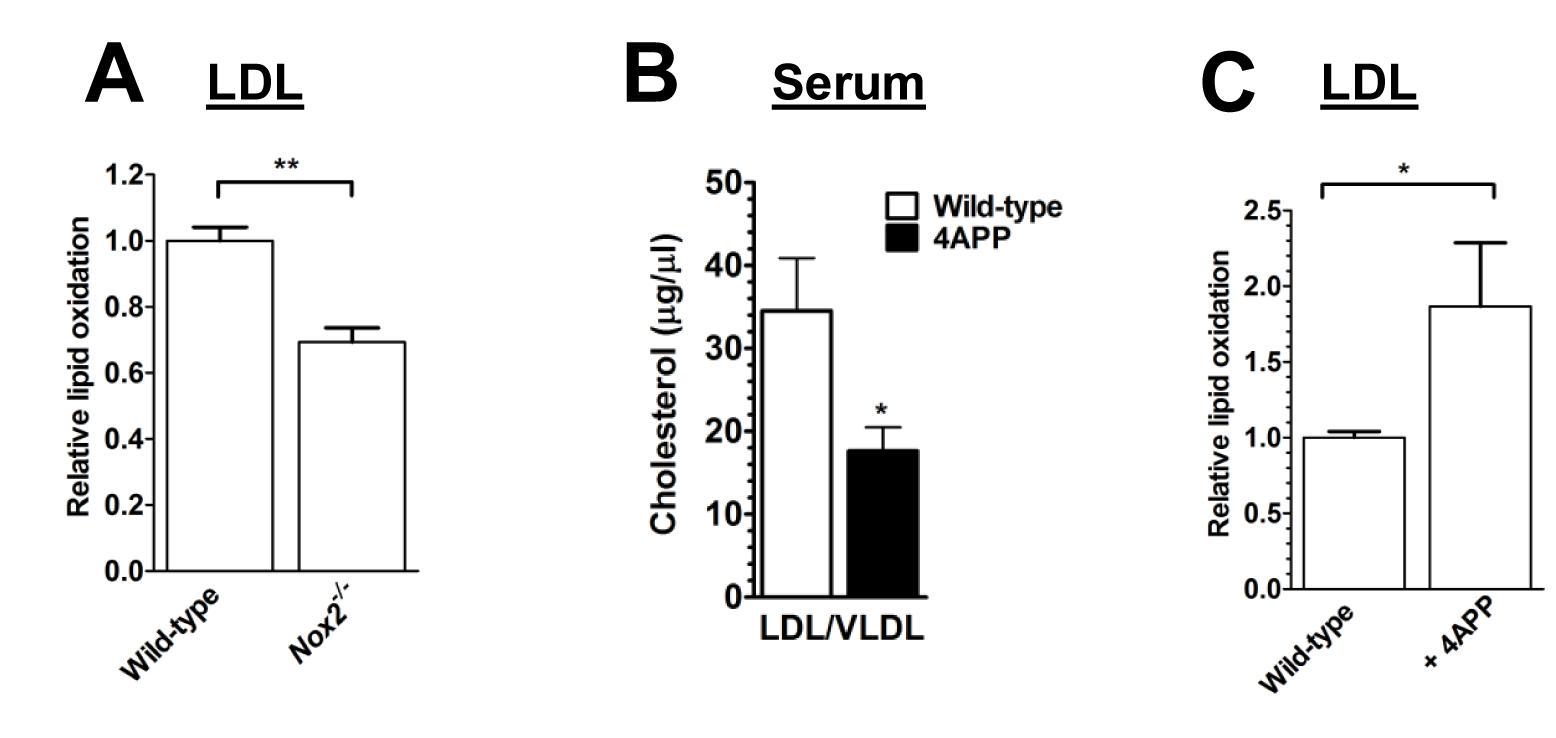

Supplement: Figure S2 — Effect of Nox2 deletion on oxidation of LDL and of 4APP on serum cholesterol associated with apoB-containing lipoprotein particles. (A) Lipid oxidation of LDL purified from the serum of Nox2−/− mice relative to LDL from wild-type mice. (B) Serum was collected from wild-type mice treated with vehicle control or 4APP and the cholesterol content of the LDL/VLDL fraction determined. (C) Lipid oxidation of LDL purified from the serum of 4APP-treated wild-type mice relative to LDL from control treated mice. Data reported as the mean ± SEM. *, p<0.05; **, p<0.01. (TIF) [file ppat.1003166.s002.tif]

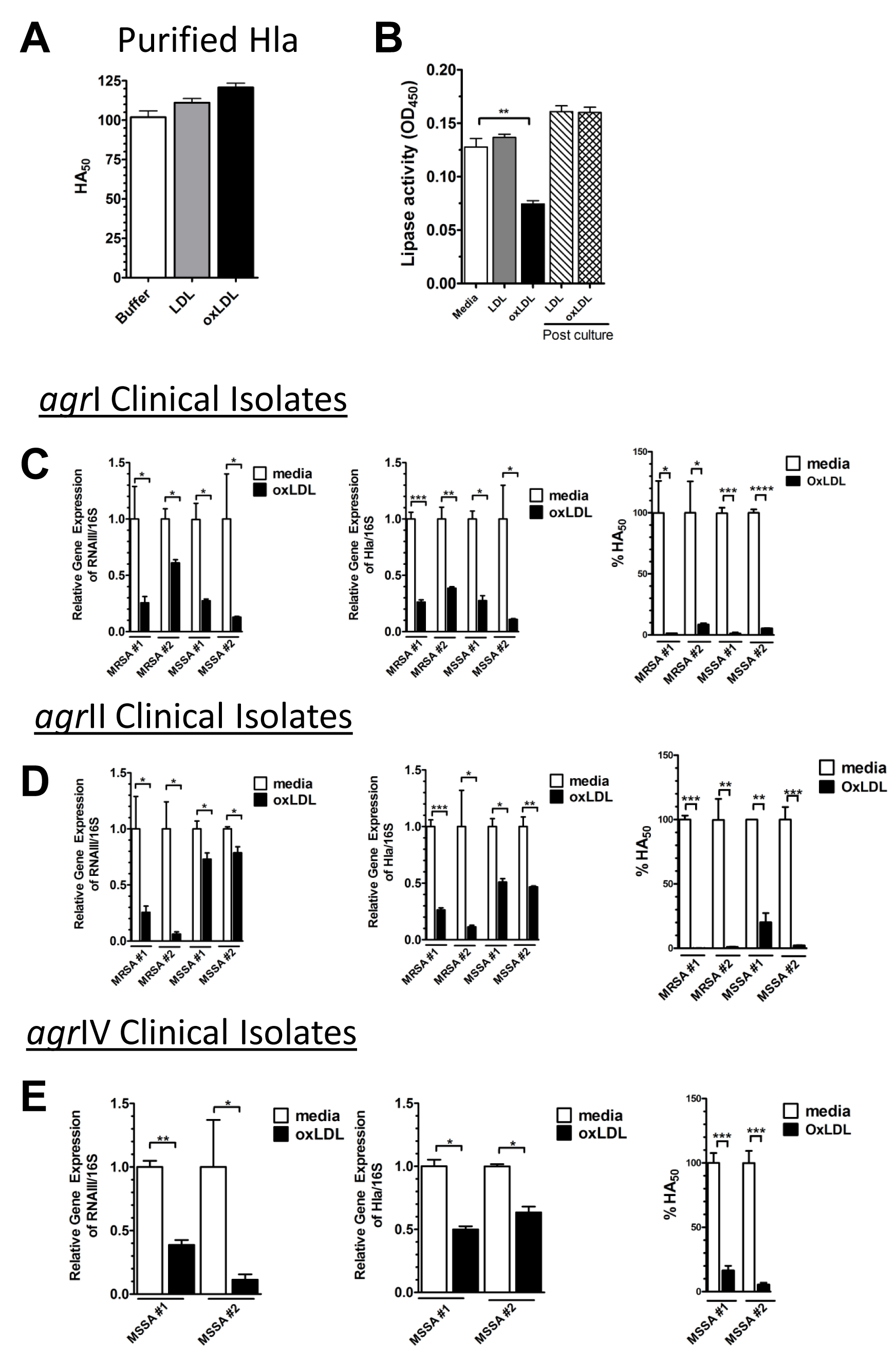

Supplement: Figure S3 — OxLDL antagonizes RNAIII transcription and agr -dependent virulence factor transcription and expression by MRSA and MSSA agr I, II and IV clinical isolates. (A) Addition of LDL or oxLDL to (A) purified Hla or (B) supernatant containing lipase has no direct impact on the activity of either virulence factor as measured by functional assay. (C–E) Clinical isolates were cultured 2 h with 50 nM AIP (agrI and II) or overnight without exogenous AIP (agrIV) plus 10 nM oxLDL or media control. Transcription of RNAIII and hla relative to 16S rRNA was measured by qRT-PCR and Hla expression was assessed by functional assay and reported as percent activity relative to media control. *, p<0.05; **, p<0.01; ***, p<0.001; ****, p<0.0001. (TIF) [file ppat.1003166.s003.tif]

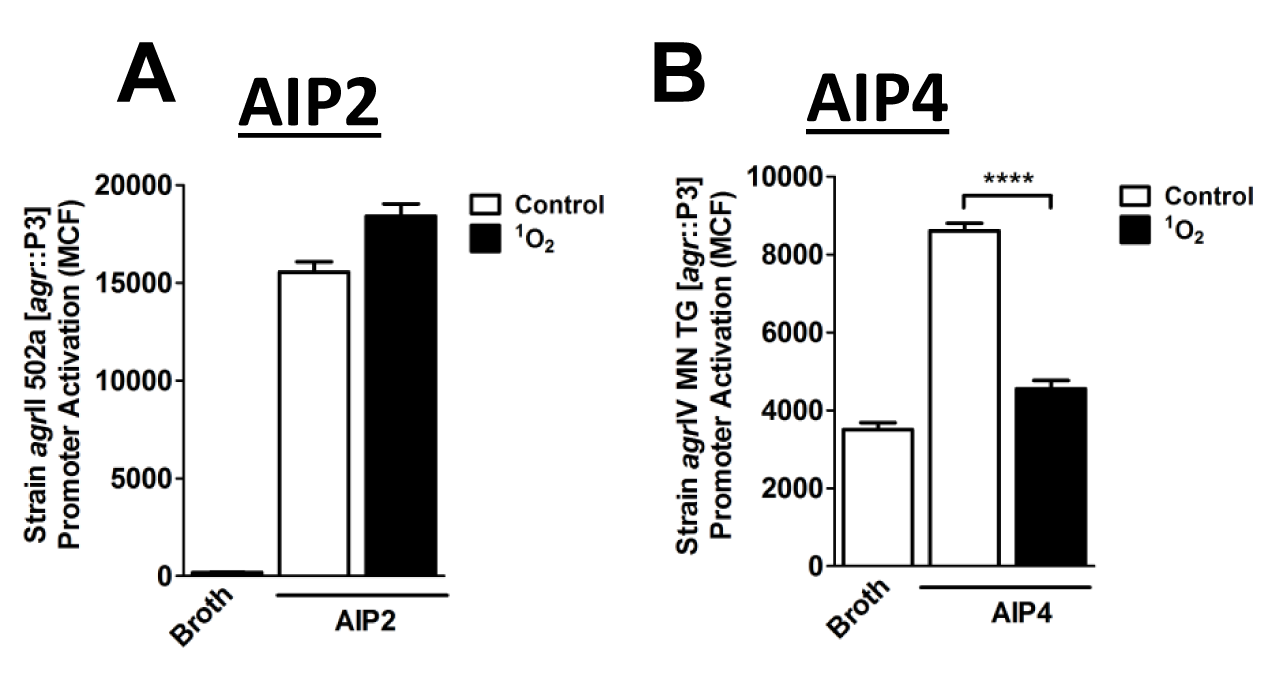

Supplement: Figure S4 — Singlet oxygen mediates oxidative inactivation of AIP4 but not AIP2. AIP2 and AIP4 were exposed to singlet oxygen by incubation with rose bengal in the presence or absence of light, prior to culture with (A) AH430 or (B) AH1872, respectively. agr::P3 promoter activation was measured by flow cytometry. Data points represent the mean ± SEM of the mean channel fluorescence. ****, p<0.0001. (TIF) [file ppat.1003166.s004.tif]

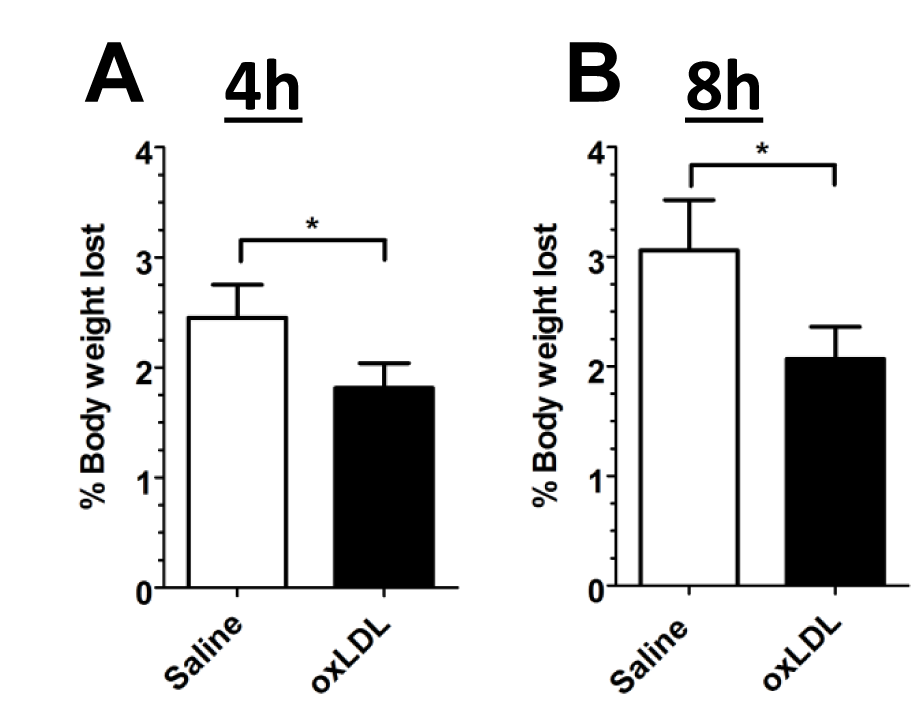

Supplement: Figure S5 — Exogenous oxLDL reduces morbidity in S. aureus agr III infected 4APP-treated Nox2−/− mice at 4 and 8 h post-infection. Air-pouches were generated on the backs of 8 to 12 week old Nox2−/− mice treated with 4APP as previously described. At time zero, 4×10^7^ cfu of early exponential phase agrIII MW2 [agr::P3-yfp] were injected into the air-pouch along with 100 nM AIP3 and either 100 nM oxLDL or saline control. Weight loss was measured at the time of infection and at (A) 4 h and (B) 8 h post-infection and is shown as percent body weight lost relative to time zero. *, p<0.05; **, p<0.01. (TIF) [file ppat.1003166.s005.tif]
